# Isolation and Characterization of Lactic Acid Bacteria and Yeasts from Typical Bulgarian Sourdoughs

**DOI:** 10.3390/microorganisms9071346

**Published:** 2021-06-22

**Authors:** Mariana Petkova, Petya Stefanova, Velitchka Gotcheva, Angel Angelov

**Affiliations:** 1 Department of Microbiology and Environmental Biotechnology, Agricultural University, 12 Mendeleev Blvd., 4000 Plovdiv, Bulgaria; marandonova@gmail.com; 2 Department of Biotechnology, University of Food Technologies, 26 Maritza Blvd., 4002 Plovdiv, Bulgaria; petya@uft-bio.com (P.S.); gotcheva_v@uft-bio.com (V.G.)

**Keywords:** Bulgarian sourdoughs, lactic acid bacteria, yeast, microbial characterization

## Abstract

Traditional sourdoughs in Bulgaria were almost extinct during the centralized food production system. However, a rapidly developing trend of sourdough revival in the country is setting the demand for increased production and use of commercial starter cultures. The selection of strains for such cultures is based on geographical specificity and beneficial technological properties. In this connection, the aim of this study was to isolate, identify and characterize lactic acid bacteria (LAB) and yeasts from typical Bulgarian sourdoughs for the selection of strains for commercial sourdough starter cultures. Twelve samples of typical Bulgarian sourdoughs were collected from different geographical locations. All samples were analyzed for pH, total titratable acidity and dry matter content. Enumeration of LAB and yeast was also carried out. Molecular identification by 16S rDNA sequence analysis was performed for 167 LAB isolates, and 106 yeast strains were identified by ITS1-5.8S-ITS2 rRNA gene partial sequence analysis. The LAB strains were characterized according to their amylolytic and proteolytic activity and acidification capacity, and 11 strains were selected for further testing of their antimicrobial properties. The strains with the most pronounced antibacterial and antifungal activity are listed as recommended candidates for the development of starter cultures for sourdoughs or other food products.

## 1. Introduction

Bread and bakery products made with sourdoughs are becoming increasingly popular in the European market. Some traditional products from this group are registered under Regulation 1151/2012 [1] with European quality labels as “protected designation of origin,” “protected geographical indication” and “traditional specialties guaranteed.”

Sourdoughs are microbial ecosystems of spontaneously formed cultures of lactic acid bacteria (LAB) and yeast in cereal flour and water matrices characterized by extremely complex dynamics of the mixed culture during the fermentation process [2,3,4,5]. The main processes occurring as a result of the metabolic activity of sourdough microflora are acidification, formation of specific flavor and dough rising [6,7,8].

As the mixed microbial cultures in the sourdoughs are formed spontaneously, the stability of the mature doughs depends on a number of factors: (1) the microflora of the flour and other ingredients used, as well as the environment; (2) the metabolic activity in the dough—production of amylolytic and proteolytic enzymes by the microorganisms, and other physiological characteristics; (3) chemical composition and enzymatic activity of the flour; and (4) technological process parameters (flour/water ratio, fermentation and storage temperature, pH and redox potential, number of backsloppings, use of starter cultures and/or baker’s yeast, etc.) [9,10,11,12,13,14]. As a result of the heterogeneity of these factors, mature sourdoughs differ in the diversity of microbial species and metabolic activities [15,16,17,18,19].

LAB and yeasts in sourdoughs use various carbohydrates and proteins, produce organic acids and contribute to flavor formation. In addition, controlled proteolysis of gluten in flour can be used to produce bread suitable for celiac disease patients and consumers with irritable bowel syndrome [20,21,22,23]. Moreover, many sourdoughs are prepared with various wholegrain flours. Due to the composition of dietary fiber and bioactive compounds in the breads and the rate of starch hydrolysis, metabolic responses positively affect postprandial glycemia, insulinemia and satiety [24].

A number of beneficial effects of sourdough fermentation are related to product quality and safety. Homopolysaccharides produced by LAB may have a favorable effect on the viscoelastic properties of the dough, the structure and shelf life of bread [6,25,26]. Acetic acid and lactic acid produced during sourdough fermentation also exhibit antibacterial and antifungal activity [27,28,29]. In addition to their effect, the production of bacteriocins helps LAB to dominate the ecosystem and may inhibit the growth of some spoilage-causing bacilli (the so-called “potato disease”) [30,31].

A number of studies have focused on the antibacterial and antifungal activity of LAB [32,33,34,35]. Magnusson and Schnurer [36] demonstrated in vitro the antifungal potential of a newly identified *Lactobacillus coryniformis* strain against different plant pathogenic, toxigenic and gushing-active *Fusarium* fungi, as well as production of the proteinaceous antifungal compound reuterin by the same strain. Lavermicocca et al. [37] reported the isolation of the antifungal compounds phenyllactic acid and 4-hydroxyphenyllactic acid from *Lactobacillus plantarum*. Gerbaldo et al. [38] showed in vitro that the growth of toxigenic storage fungi was restricted by two LAB strains *Lb. rhamnosus* L60 and *Lb. fermentum* L23 and attributed this to the combined effect of lactic acid and bacteriocin. Strains *Lb. plantarum* F1 and *Lb. brevis* OG1 isolated from Nigerian fermented food products produced bacteriocins that had a broad spectrum of inhibition against pathogenic, food spoilage organisms and various lactic acid bacteria [39]. Muthusamy et al. also demonstrate that the antifungal activity of LAB is attributed to the production of different inhibitory compounds—organic acids (mainly lactic and acetic acid), hydrogen peroxide and other antimicrobial compounds such as bacteriocins [40].

Studies of species diversity in the sourdoughs and population dynamics during fermentation play an important role in understanding the complex fermentation process as well as producing standardized, high-quality end products [41,42,43]. A wide variety of molecular techniques for the identification of LAB and yeast in sourdoughs have been elaborated, such as random amplification of polymorphic DNA (RAPD) [44,45], restriction fragment length polymorphism (RFLP) [46], determination of chromosome polymorphism by pulse-field gel electrophoresis (PFGE), as well as denaturing gradient gel electrophoresis (DGGE) [47]. The 16S rDNA-based methods are widely used to identify genetic relationships among bacteria [48]. With regards to yeast in sourdoughs, the identification of new isolates to the species level is achieved by DNA sequencing and analysis of the ITS-5.8S-ITS2 region because of the high level of interspecific sequence variability of ITS [49,50].

In recent years, the use of metagenomic approaches, including innovative mass sequencing multi-amplitudes of 16S/ITS/28S rRNA is applied to fully characterize the ecosystems of sourdoughs. The use of mass parallel sequencing gives the possibility of sequencing and annotating millions of sequences and thus identifying hundreds of microorganisms simultaneously in the acidic ecosystem of the dough [51,52,53,54].

The increasing scale of sourdough production sets the demand for controlled processes to ensure continuous product quality. Therefore, the application of selected starter cultures is essential to ensure the sustainable production of quality sourdough products. Various selection criteria are applied to develop industrial starter cultures, such as acidification, resistance to microbial competitors, adaptation to environmental conditions, antifungal activity, aroma development, improvement of dough structure and functional properties contributing to improving the health of the consumers [6,23,29,55]. However, the starter cultures used are often selected only on the basis of a particular property (e.g., acidification or flavor formation) and are not sufficiently competitive [5,56].

In Bulgaria, the traditional preparation of sourdoughs was almost eliminated during the long years of the centralized political and economic system and, respectively, food production system. Sourdough products are currently prepared in isolated households or small bakeries, still preserving old practices, but scientific knowledge about them is lacking. However, there is a trend of the revival of sourdough technology due to the consumers’ increased interest in sourdough bread and bakery products perceived as “healthier” and having unique taste, aroma and structure, as well as a significantly longer shelf life.

With regards to this trend, the aim of the present study was to isolate, identify and characterize lactic acid bacteria and yeasts from typical Bulgarian sourdoughs for a further selection of strains for commercial sourdough starter culture development.

## 2. Materials and Methods

### 2.1. Sourdoughs

Samples from 12 sourdoughs used for the manufacture of typical Bulgarian bread were collected from different regions. Information about the geographical origin, ingredients and preparation methods is presented in Table 1. All samples were taken at the end of the final backslopping and were transported and stored at 4 °C before analyses.

### 2.2. Physico-Chemical Characterization of Sourdoughs

All samples were analyzed for total titratable acidity (TTA), pH and dry matter content. TTA was analyzed by titration with 0.1 N NaOH to pH of 8.4, and pH was measured by a pH meter Mettler Toledo FiveEasy FE20. Dry matter was determined by drying 5 g of each sample at 100–105 °C to constant weight.

### 2.3. Lactic Acid Bacteria and Yeast Enumeration and Isolation

Decimal dilutions of the sourdough samples were prepared with peptone water (1% (*w/v*) peptone and 0.9% (*w/v*) NaCl). Four culture media were used to determine the total viable counts of LAB in the samples: MRS (Merck, Darmstadt, Germany), MRS-5 (Meroth et al., 2003), M17 (Merck, Darmstadt, Germany) and M17-glucose (containing 0.5% *w/v* glucose instead of lactose). All media were supplemented with cycloheximide (0.1 g/L). The plates were incubated under anaerobic conditions (AnaeroGen, Oxoid Ltd., Hampshire, UK) at 37 °C for 48 h. From each medium, a number of colonies equal to the square root of the total number recorded in Petri dishes with 15 to 300 CFUs were randomly selected for isolation. The isolates were examined microscopically and tested by Gram staining and catalase reaction. Pure cultures were further obtained from the isolates that were Gram-positive, catalase-negative, nonmotile rods and cocci after sub-culturing in the respective liquid medium and streaking on agar media. Stock cultures were stored in Microbank™ vials (Pro-Lab Diagnostics Inc.,Richmond Hill, ON, Canada) at −70 °C. The total viable counts of yeasts in the sourdough samples were estimated on malt extract agar (MEA) and Sabouraud dextrose agar (SDA) (Merck, Darmstadt, Germany) supplemented with chloramphenicol (0.1 g/L) at 30 °C for 48 h. From each medium, a number of colonies equal to the square root of the total number recorded in Petri dishes with 15 to 300 CFUs were randomly selected for isolation. Morphological characterization of the yeast isolates was performed by microscopic analysis. The isolates were sub-cultured in malt extract broth and streaked onto the same agar media. Stock cultures were stored in Microbank™ vials (Pro-Lab Diagnostics Inc.) at −70 °C.

### 2.4. Molecular Identification of Lactic Acid Bacteria by 16S rDNA Sequence Analysis

The total genomic DNA from LAB strains was extracted from overnight cultures grown in MRS. DNA extraction of LAB was conducted using HigherPurity™ Bacterial Genomic DNA Isolation Kit (Canvax Biotech, S.L., Cordoba, Spain), according to the manufacturer’s instructions. The quality and concentration of DNA extracts were assessed by determination of absorbance at 260 nm and 280 nm (Shimadzu UV-VIS, Shimadzu Corporation, Japan). LAB identification was performed by PCR amplification of the 16S rRNA gene with conventional PCR (2720 Thermal Cycler, Applied Biosystems, Waltham, MA, USA) and sequencing of the PCR products. The oligonucleotide primers used in this study were forward primer LacbF (5′-TGCCTAATACATGCAAGT-3′) and reverse primer LacbR (5′-CTTGTTACGACTTCACCC-3′) [14], obtained from Metabion (Martinsried, Germany). The PCR analysis was performed in final reaction volumes of 20μL containing 1μLof DNA (50 ng), 0.5 μM of each primer and 8μLof Red-Taq DNA Polymerase Master Mix (Canvax Biotech, S.L., Spain). The parameters of amplification were the following: initial denaturation at 94 °C for 5 min, 35 cycles of 1 min at 94 °C, 45 s at 50 °C and 2 min at 72 °C, and final extension at 72 °C for 5 min. Further, the obtained amplicons were stained with Safe View (NBS Biologicals, Huntingdon, England) and separated on 1% agarose gel carried out in 0.5x TBE buffer (45 mmol/L Trisborate and 1 mmol/L EDTA) for 60 min at 100 V, using a VWR Mini Electrophoresis system (VWR, Darmstadt, Germany) and MiniBis Pro (DNR Bio-Imaging Systems, Israel) for gel visualization. The PCR products (approximately 1200 bp) were cut out from the gel and purified with Clean-Easy™ Agarose Purification Kit (Canvax Biotech, S.L., Spain). Sequencing of the PCR products was performed by MicrosynthSeqlab (Göttingen, Germany). The resulting sequences were analyzed using BLAST algorithm [57] and compared with the nucleotide sequences in the gene bank database (www.ncbi.nlm.nih.gov, accessed on 8, 10 and 24 March 2021). The phylogenetic tree was obtained by means of the unweighted pair group method using the arithmetic average (UPGMA) clustering algorithm [58] and CLC Genomics Workbench 20.0 (https://digitalinsights.qiagen.com).

### 2.5. Molecular Identification of Yeast by ITS1-5.8S-ITS2 rRNA Gene Sequence Analysis

Prior to DNA extraction, yeast strains were cultured for 24 h on a YMA medium. Yeast genomic DNA was extracted by Higher-Purity™ Yeast Genomic DNA Isolation Kit (Canvax Biotech, S.L., Spain). The quality and concentration of DNA extracts were determined by spectrophotometric measurements using Shimadzu UV-VIS spectrophotometer (Shimadzu Corporation, Kyoto, Japan). The ITS-5.8S-ITS2 region was amplified by forward primer ITS 4 (5′-TCCTCCGCTTATTGATATGC-3′) and reverse primer ITS 5 (5′-GGAAGTAAAAGTGCTAACAAGG-3′) [59], obtained from Metabion (Martinsried, Germany). The PCR reaction mix contained 1μLof DNA (50 ng), 0.5 μM of each primer and 8μLof Red-Taq DNA Polymerase Master Mix (Canvax Biotech, S.L., Spain) in total volume of 20μL. The amplification was carried out in a PCR 2720 Thermal Cycler (Applied Biosystems, USA) using the following program: initial denaturation at 95 °C for 10 min, followed by 35 cycles of denaturing at 94 °C for 1 min, annealing at 52 °C for 1 min, extension at 72 °C for 1 min and final extension at 72 °C for 7 min. PCR products were visualized in 1% agarose gel stained with SafeView (NBS Biologicals, Huntingdon, England) at 100 V for 50 min using VWR Mini Electrophoresis System (VWR, Germany) and MiniBis Pro (DNR Bio-Imaging Systems, Israel) for gel visualization. The PCR products (approximately 700 bp) were cut out from the gel and purified with Clean-Easy™ Agarose Purification Kit (Canvax Biotech, S.L., Spain). Sequencing of the PCR products was performed by MicrosynthSeqlab (Göttingen, Germany). The resulting sequences were analyzed using the BLAST algorithm [57] and compared with the nucleotide sequences in the gene bank database (www.ncbi.nlm.nih.gov, accessed on 21 and 24 March 2021). The phylogenetic tree was obtained by the unweighted pair group method using the arithmetic average (UPGMA) clustering algorithm [58] and CLC Genomics Workbench 20.0 (https://digitalinsights.qiagen.com).

### 2.6. Screening of the LAB Isolates for Amylolytic and Proteolytic Activity and Acid-Producing Capacity

The amylolytic activity of the LAB strains was assessed by the agar-diffusion method [60]. All isolated were subjected to qualitative screening for amylolytic activity by cultivation on solid media containing starch as a major carbon source. The agar-diffusion method was performed with Starch agar (g/L): wheat starch—10; peptone—5; yeast extract—5; MgSO_4_·7H_2_O—0.25; FeSO_4_·7H_2_O—0.01; agar −15; pH 6.8 ± 0.2. In each agar Petri dish, four wells with 10 mm diameter were made, and the wells were inoculated with 100 μL 24 h MRS medium-cultivated bacterial suspensions. After incubation at 37 °C for 48 h, the plates were treated with iodine solution to form a blue-colored starch-iodine complex. The diameter of the transparent zones around the colonies of amylolytic bacteria was measured after color was left to develop for 5 min. The amylolytic index (AI) was calculated as the ratio R/r, where R was the diameter of the entire clear zone, and r was the diameter of the agar well with the LAB colony [61]. The proteolytic activity of the LAB isolates was analyzed by the method of Hébert et al. [62]. The analysis was performed on skim milk agar (casein 0.5%, yeast extract 0.25%, dextrose 0.1%, skimmed milk powder 2.5% and agar 1.5%). In each agar Petri dish, four wells with 10 mm diameter were made, and the wells were inoculated with 100 μL 24 h MRS medium-cultivated bacterial suspensions from each isolate. Results were observed after 48 h incubation at 37 °C. The protein hydrolysis index (PHI) was calculated as the ratio R/r, where R was the diameter of the entire clear zone, and r was the diameter of the agar well with the LAB colony [61]. The acid-producing capacity of the LAB isolates was tested by pH measurement (Mettler Toledo FiveEasy FE20) at the beginning and end of 24 h cultivation in MRS medium.

### 2.7. Antimicrobial Activity of the LAB Strains

The antibacterial activity of the isolated lactic acid bacteria was tested by the agar well diffusion method of Yang et al. [63]. Cell-free supernatants (CFSs) were obtained by centrifugation of 24 h MRS-broth cultures of the tested LAB. The CFSs were consecutively subjected to the following treatments: adjustment of pH to 6.5, boiling for 20 min, addition of catalase (5 mg/mL) (Merck, Darmstadt, Germany) and treatment with trypsin (1 mg/mL) (Merck, Darmstadt, Germany). The following test microorganisms were used in the assay: *Bacillus subtilis* NBIMCC 3562, *Staphylococcus aureus* NBIMCC 3081 and *Salmonella enterica* NBIMCC 8691. After inoculation of the 5 mm wells with 100 μL of the respective CFS, the plates were incubated at 37 °C for 48 h and the areas of growth inhibition area were measured. Screening for antifungal activity of the LAB strains was carried out against spoilage fungi by the agar well diffusion method using CFSs obtained and treated as described above. Peptone Yeast Extract Agar (Merck, Darmstadt, Germany) was inoculated with 1 × 10^4^ spores of the mold cultures *Penicillium chrysogenum* NBIMCC 129, *Fusarium graminearum* NBIMCC 2294, *Rhizopus stolonifer* NBIMCC 130 and *Aspergillus nidulans* NBIMCC 116. After inoculation of the 5 mm wells with 100 μL of the respective CFS, the plates were incubated at 28 °C for five days and the areas of growth inhibition area were measured.

### 2.8. Nucleotide Sequence Accession Number

16s rRNA gene sequences of LAB were deposited in GenBank and assigned the following accession numbers: MW774565-MW77456, MW694895, MW685418-MW685439, MW683129-MW683203, MW682282-MW682287, MW682219-MW682281. The yeast ITS5 and ITS2 ribosomal regions determined in this study were deposited in GenBank and assigned the accession numbers as follows: MW774571-MW774573, MW756316-MW756319, MW756228-MW756315, MW756212-MW756227.

### 2.9. Statistical Analysis

All experiments were carried out in triplicate. Data were subjected to one-way ANOVA; pair comparison of treatment means was obtained by Tukey’s test (Statistica 8.0 software package). Differences were reported at a significance level of *p* ≤ 0.05.

## 3. Results and Discussion

### 3.1. Physico-Chemical Characterization of Sourdoughs

Samples of a total of 12 typical Bulgarian sourdoughs, all produced without the addition of commercial yeast, were collected from 5 bakeries and 1 household in Bulgaria. The selection of sourdoughs was based on the difference of geographical locations, the difference in raw materials—white and whole-grain wheat (*Triticum aestivum*) flours, flours from rye (*Secale cereale*), chickpea (*Cicer arietinum*), einkorn wheat (*Triticum monococcum*)—used as single raw materials or in combinations, the addition of NaCl and preparation methods—fermentation time and temperature, number of backsloppings (Table 1). Since the sourdough tradition was almost lost during the past 60 years and the interest toward sourdough production was revived only in the past 10 years, most sourdoughs found were recently initiated (1–2 years) and only a few had been sustained for several years.

All samples were analyzed for pH, total titratable acidity (TTA) and dry mater content (DM) of the samples are listed in Table 2. The values of pH ranged from 3.58 (sample 10B4) to 5.11 (06SE). Considering that good quality sourdoughs have pH within 3.5–4.1 [7], nine of the analyzed samples were with pH values within this interval, with an average of pH 3.69. These values are similar to the pH interval of 3.74–4.28 and 3.41–3.70 reported for Italian and French sourdoughs, respectively [14,64].

Dry matter content ranged from 33.01 (sample 10B4) to 58.16 (05S) depending on the sourdough recipe. Although the sourdough sample with the lowest DM (33.01%) was also with a very low pH value (3.58), DM was not the main factor affecting sourdough acidification. The lack of direct correlation between DM and pH may be explained with the effects of the flour types used, diversity of the sourdough microbiota and the baker’s practices [2].

TTA was also analyzed as an important indicative parameter for fermentation processes resulting in organic acid formation. In general, TTA of good quality sourdoughs ranges from 14 to 16 mL [6]. For eight of the studied sourdoughs (01M, 02P1, 04P3, 07B1, 09B3, 10B4, 11R1 and 12R2), TTA was within this interval, and in most of the cases TTA formation could be linked to the dry matter content. As an example, the highest acid formation was observed in the sample with the lowest dry matter (10B4), and the second-highest DM content sample had the lowest TTA value (sample 06SE). However, other factors are also important determinants of the organic acid, such as the composition of the fermenting matrix, physiology of the sourdough microbiome, fermentation conditions, etc. [16].

### 3.2. Enumeration of LAB and Yeasts

Microbial characterization of the sourdoughs was carried out by the determination of lactic acid bacteria (LAB) and yeast counts. Four different agar media (MRS, MRS5, M17 and M17 G) were used for the isolation and enumeration of the presumptive LAB in the samples to ensure obtaining representative results (Table 2). The observed LAB counts ranged from 1.4 × 10^4^ (sample 06SE, MRS5) to 9.7 × 10^9^ (sample 10B4, MRS), and 62.5% of all samples had more than 10^8^ cfu/g presumptive LAB. The differences in LAB counts on different media may be attributed to the variations of the carbon sources and the different assimilation capacities of the sourdough microorganisms [65,66]. When comparing the performance of the culture media, MRS and MRS5 medium harbored the highest cfu numbers, which confirms other data from studies on bacterial diversity of sourdoughs. Some researchers also found higher LAB counts on MRS than on MRS5 [14,67].

Yeast counts in the sourdoughs varied from 1.0 × 10^4^ (sample 06SE, SA) to 5.3 × 10^9^ (sample 05S, SA), with 29.2% of the samples showing content of more than 10^7^ cfu/g. Analysis of most samples resulted in similar cfu numbers on both culture media. The approximate ratio between LAB and yeasts of the studied sourdoughs was between 100:1 and 10:1. Similar ratios were reported by other authors as well—10:1 in French organic sourdoughs [68], and 100:1 in sourdoughs type 1 [69,70]. Sourdough sample 06SE was the only exception with an LAB to yeast ratio of 1:1. This microbial ratio logically resulted in the lowest organic acid accumulation in the sample (highest pH value of 5.11 and lowest TTA of 8.2) (Table 2). Again, direct connection with dry matter content was not found.

### 3.3. Molecular Identification of LAB by 16S rDNA Sequence Analysis

The presumptive LAB isolates selected from the 4 agar media amounted to 215, of which 167 were proved to be Gram-positive and catalase-negative rods or cocci. The cultures were purified and were further identified by PCR amplification of the 16S rRNA gene and sequencing of the PCR products. This approach has become a major tool in the determination of relationships between bacteria, and it is widely used for identification purposes [14].

The obtained sequences were processed and subjected to BLAST analysis. Results showed the presence of three genera—*Lactobacillus* (51.5%), *Pediococcus* (44.9%) and *Enterococcus* (3.6%) (Table S1). The most predominant species found were *Lactobacillus plantarum* (35.9% of all LAB isolates) and *Pediococcus pentosaceus* (34.7%). The results corresponded to the findings of Robert et al. [71]. The authors identified six genera of LAB in traditional French sourdoughs and reported similar percentages of the most predominant species—*Lactobacillus* (39%) and *Pediococcus* (38%). In contrast to the same study, *Lactobacillus brevis* and *Pediococcus acidilactici* were also common in Bulgarian sourdoughs—13.2% and 9.6%, respectively. *Lactobacillus plantarum*, *Lactobacillus plantarum* subsp. *plantarum*, *Enterococcus faecium*, *Enterococcus durans* and *Pediococcus parvulus* were also found in the sourdough samples. Strains of *Enterococcus faecium* isolated from sourdoughs were associated with safety and probiotic potential by some authors [72]. All analyzed strains were identified with a high level of confidence—*Lactobacillus* spp. (97.57–100%), *Pediococcus* spp. (97.20–100%) and *Enterococcus* spp. (99.07–100%) (Table S1).

The relationship between the identified LAB was established by using cluster analysis. The obtained phylogenetic tree is presented in Figure S1. All strains of *Lactobacillus plantarum* and *Lactobacillus plantarum* subsp. *plantarum* were closely related to each other. They also show a high level of similarity with some representatives of *Lactobacillus brevis.* These results are in agreement with other studies [14,71]. The strains from *Enterococcus* species were grouped separately. The representatives of *Pediococcus* species were also grouped in a different cluster. The clustering of the strains was not related to the products they were isolated from.

### 3.4. Molecular Identification of Yeasts by ITS1-5.8S-ITS2 Region Sequence Analysis

The Internal Transcribed Spacer (ITS) regions of yeast ribosomal DNA (rDNA) are highly variable sequences of great importance in distinguishing yeast species by PCR analysis [50]. A total of 106 yeast isolates obtained from Bulgarian sourdoughs were subjected to ITS1-5.8S-ITS2 rRNA gene partial sequence analysis. Results show that the analyzed yeast strains belonged to five genera—*Saccharomyces*, *Kazachstania*, *Pichia*, *Kluyveromyces* and *Yarrowia* (Table S2). *Saccharomyces cerevisiae* was the most predominant species (86.8%) and it was found in all analyzed samples. These results were in agreement with other studies [73,74,75]. Five strains were identified as *Kazachstania barnettii*, one strain was *Kazachstani ahumilis*, and four strains belonged to *Kluyveromyces marxianus*. The identification was made with a high percentage of confidence (98–100%). The presence of *Kazachstania barnettii* in sourdoughs was also reported by Minervini et al. [14]. Very few authors have so far reported the occurrence of *Y. lipolytica* in sourdoughs, but it is found as a minor but quite regular part of the microbiota of other fermented food products of plant origin. The presence of *Y. lipolytica* in sourdoughs is always along with *S. cerevisiae*, most probably due to its lacking of fermentative metabolism [76,77].

All yeast isolates from several sourdoughs (05S, 08B2, 10B4, 11R1 and 12R2) were identified as *Saccharomyces cerevisiae*, which could be attributed to the limitations of the culture-dependent method applied.

A phylogenetic tree of the identified yeast strains is presented in Figure S2. The results show that all yeast species found have a high level of similarity, which is in agreement with the results of Peterson and Kurtzman [78]. The representatives of different species were separated in some clusters, but the species are nevertheless closely related.

### 3.5. LAB Selection

The main selection criteria for LAB strains for sourdough applications is their ability to assimilate starch as a single carbon source by the production of amylolytic enzymes, ability to degrade proteins and acidification of the dough matrix [14,79]. The results presented in Table S3 show that more than 25% of the isolated LAB have good amylolytic activity. The amylolytic index (AI) of 22.75% of the strains was between 4 and 5, and four strains showed AI values > 5. The highest AI was exhibited by representatives of *Pediococcus pentosaceus*, *Lactobacillus plantarum*, *Lactobacillus brevis*, *Enterococcus faecium* and *Enterococcus durans*, with *Pediococcus pentosaceus* 12R2192 reaching AI of 5.79. High amylolytic activity of Bulgarian strains isolated from sourdoughs was observed in our previous studies as well [48,80]. It is interesting to note that despite the low number of identified *Enterococcus* spp. representatives, five out of the six strains had amylolytic indexes above 4.

During sourdough fermentation, LAB may release small peptides and free amino acids as a result of their strain-specific proteolytic systems. The proteolytic activities of LAB are important not only for their development in a cereal matrix but also for the potential to decrease gluten and other anti-nutritional factors and release bioactive peptides and essential amino acids that contribute to human well-being [53]. Proteolytic activity is a very important starter culture characteristic since the extent of proteolysis during lactic acid fermentation is strongly related to the structure, the organoleptic characteristics, the digestibility and the shelf life of the sourdoughs [17]. In the present study, the isolated LAB strains were also characterized with regards to their proteolytic activity. Results show the strong proteolytic activity of 70.66% of the isolates, with a protein hydrolysis index (PHI) of 4–5. The PHI values observed for 25.15% of the strains range between 5 and 6, and the strain *Pediococcus pentosaceus* 12R2192 reached a PHI of 6.68. The analysis demonstrated that all LAB strains isolated from the Bulgarian sourdoughs were able to hydrolyze proteins in an agar medium.

Acid-producing capacity is a major property assessed in strain selection for starter cultures since it determines the fermentation time, the physico-chemical and organoleptic characteristics of the product, as well as its shelf life. In the current study, almost 30% of the isolated LAB strains showed high acidification capacity, with estimated differences between pH values at inoculation and at the end of the fermentation higher than 1.7. The numbers of the isolates with Δ pH of 1.7–1.8 and 1.8–1.9 were similar and represented 16.77 and 11.97% of the total identified LAB, respectively. The representatives of *Lactobacillus plantarum* subsp. *plantarum* showed significantly lower acidification capacity, while the highest acid-producing ability (1.91 ± 0.02) was observed for strain *Lactobacillus brevis* 07B198.

Based on comparative analysis of the results from the above three criteria, the following strains were selected for further research for potential food and biotechnology applications: *Pediococcus pentosaceus* 07B1109, *Pediococcus pentosaceus* 12R2192, *Lactobacillus plantarum* 08B212, *Lactobacillus plantarum* 08B217, *Lactobacillus brevis* 01M22, *Lactobacillus brevis* 04P3167, *Lactobacillus brevis* 06SE269, *Lactobacillus brevis* 07B198, *Enterococcus faecium* 12R226, *Enterococcus faecium* 12R232 and *Enterococcus durans* 09B374.

### 3.6. Antibacterial Activity of the LAB Strains

Sourdoughs are usually prepared by hand and there is a high risk of dough contamination with microorganisms from the environment and the personnel. *B. subtilis* is a typical contamination issue for the bread industry, especially during the warm seasons, since it decreases bread shelf life by causing the so-called “rope” of bread (stringy strands of mucilage, stickiness, unpleasant flavor and taste) [81], while *Staphylococcus aureus* (Gr+) and *Salmonella enterica* (Gr−) are common pathogens which may be transferred from the personnel during raw materials handling [82].

LAB are capable of producing antimicrobial compounds such as organic acids, H_2_O_2_ and bacteriocins, which is the basis of the natural biopreservation of fermented foods [83]. Therefore, the use of sourdough starter cultures able to inhibit contaminating spoilage and pathogenic bacteria is an important strategy for ensuring the quality and safety of sourdough products.

The antibacterial activity of 11 LAB strains isolated from Bulgarian sourdoughs against the three bacterial species related to bread quality and safety was determined by the well diffusion method after various treatments of cell-free supernatants to reveal the nature of this activity. The applied treatments included pH adjustment to evaluate the role of organic acids, boiling for 20 min and use of trypsin to assess the inhibitory activity of potential bacteriocins of protein nature, and catalase treatment to assess the effect of H_2_O_2_ [84].

**Figure 1:**
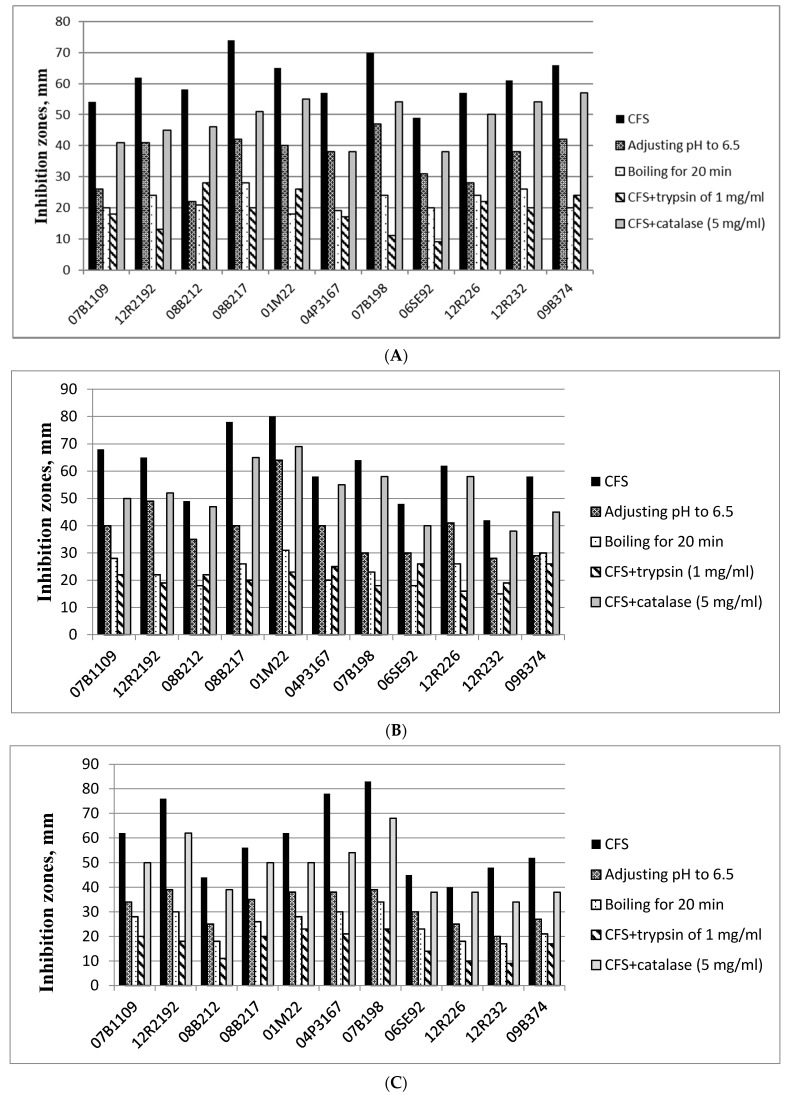
Antibacterial activity of selected LAB from Bulgarian sourdoughs against *Bacillus subtilis* NBIMCC 3562 (**A**), *Staphylococcus aureus* NBIMCC 3081 (**B**) and *Salmonella enterica* NBIMCC 8691 (**C**).

The ropiness-causing *B. subtilis* was inhibited by all the examined LAB. The highest inhibitory activity of the SFCs towards *B. subtilis* was observed for strains *Lb. plantarum* 08B217 (74 mm zone) and *Lb. brevis* 07B198 (70 mm zone), followed by *Enterococcus durans* 09B374 and strain *Lb. brevis* 01M22 (66 and 65 mm zones, respectively) (Figure 1A).

Adjusting pH from 3.9–4.4 to 6.5 led to a significant decrease (by 33 to 52%) of the antimicrobial activity of the isolates. The highest reduction was observed for strains 08B212, 07B1109 and 12R226—62%, 52% and 51%, respectively. These findings confirm that the antibacterial effect of the strains is mostly attributed to organic acid production. On the other hand, boiling of CFS, which aimed to denature potential protein compounds with antibacterial activity, resulted in an even higher decrease in inhibition of *B. subtilis* by all LAB strains, which indicates that such compounds contribute more significantly to the antibacterial activity of the strains compared to organic acid production. Todorov and Dicks [85] reported that bacteriocin ST44AM remained stable at 100 °C for 120 min, but the activity of this bacteriocin was reduced after exposure at 121 °C for 20 min. Similar results were reported for a bacteriocin produced by *Lactobacillus* CA44 [86] and also thuricin 7 from *B. thuringiensis* BMG1.7 [87].

In the present study, trypsin treatment of the CFS resulted in an even bigger decrease of the antimicrobial effect for all strains except for 08B212 и 01M22. These observations also suggest that the inhibition of the test hygiene indicators/pathogens is aided by protein components. The addition of catalase also affected the growth of *B. subtilis* compared to the controls (by approximately 11 to 33%), but its effect was much weaker than the effect of organic acids and the protein antimicrobial compounds (Figure 1A).

The highest antibacterial activity of the LAB cell-free supernatants towards *S. aureus* was observed again for *Lb. brevis* 01M22 (80 mm zone) and *Lb. plantarum* 08B217 (78 mm zone), followed by strains *Pediococcus pentosaceus* 07B1109 and 12R2192 (with 68 and 65 mm zones) (Figure 1B).

With regards to inhibition of *S. enterica*, the best results were registered for strains *Lb. brevis* 07B198 (83 mm zone), *Lb. brevis* 04P3167 (78 mm) and *P. pentosaceus* 12R2192 (76 mm zone) (Figure 1C).

Comparison of inhibitory activity against the two pathogens shows that boiling and trypsin treatment of the strains supernatants had a much more significant effect on the inhibition than pH adjustment and catalase treatment. Boiling decreased inhibitory activity towards *S. aureus* and *S. enterica* by 48–67% and 49–66%, respectively, and the effect of trypsin treatment of the supernatants decrease the pathogen inhibition by 55–74% for *S. aureus* and by 63 to 81% for *S. typhimurium* (Figure 1B,C). This observation clearly indicates that the antibacterial activity against *S. aureus* and *S. enterica* is mostly due to the production of antimicrobial compounds of protein nature. The treatment of the CFSs with catalase affected the inhibition activity less than the treatments with high temperature and trypsin, which again confirms that the inhibitory activity of the studied LAB strains is mainly attributed to protein substances.

Yang et al. [63] carried out a study on the antimicrobial effect of eight LAB isolates producing bacteriocin or bacteriocin-like substances on several Gram-positive, Gram-negative bacteria and fungi. They reported that the untreated CFS inhibited all test bacteria and fungi except for *E. coli*, but pH neutralization and H_2_O_2_ elimination resulted in the loss of the inhibitory effect towards almost all test microorganisms.

Moreover, the obtained results show that several of the isolated LAB strains—*Lb. brevis* 01M22, *Lb. brevis* 07B198, *Lb. plantarum* 08B217 and *P. pentosaceus* 12R2192—were repeatedly most active against the test spoilage and pathogenic bacteria, making them good candidates for sourdough starter culture development.

### 3.7. Antifungal Activity of the LAB Strains

**Figure 2:**
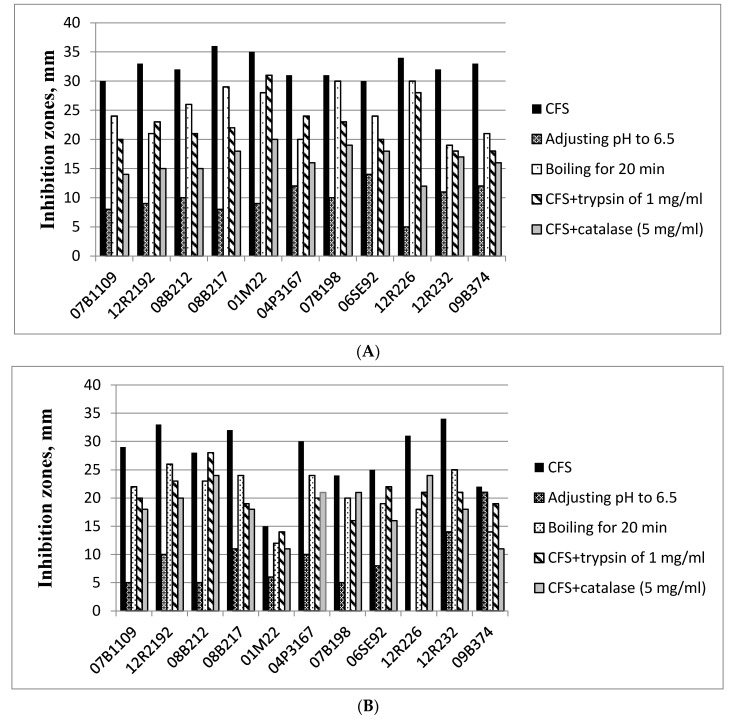

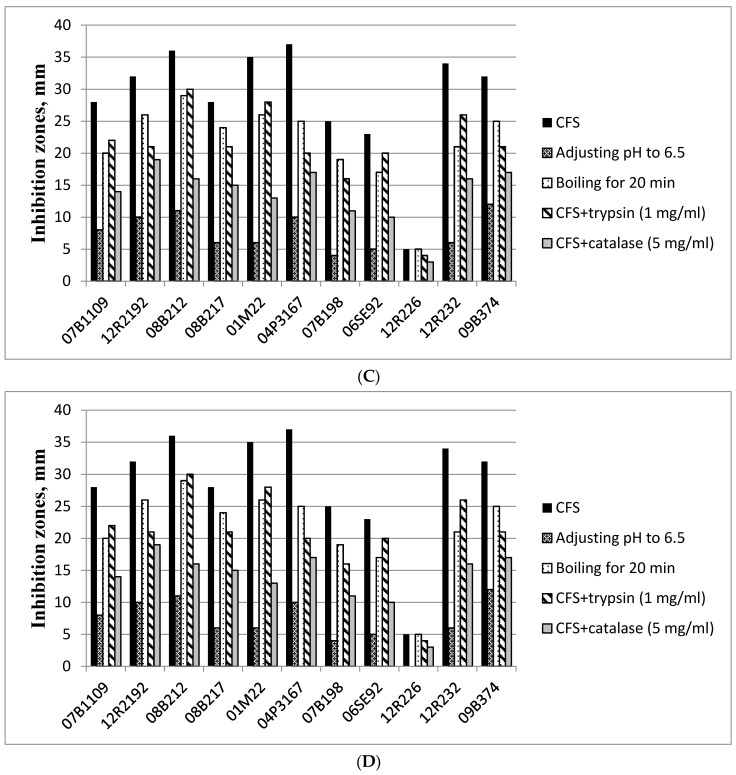
Antifungal activity of selected LAB from Bulgarian sourdoughs against *Penicillium chrysogenum* NBIMCC 129 (**A**), *Fusarium graminearum* NBIMCC 2294 (**B**), *Rhizopus stolonifer* NBIMCC 130 (**C**) and *Aspergillus nidulans* NBIMCC 116 (**D**).

The potential application of LAB as biopreservation agents in various branches of the food industry has received much attention in the past 10 years. Despite the accumulated data, there is still much to be studied in terms of strain characterization, active compounds and concentrations, strain-matrix effects, processing effects, etc. In this connection, the antifungal activity of the LAB strains isolated from Bulgarian sourdoughs was also tested in the present study against representatives of the most common cereal-contaminating fungi—*Penicillium chrysogenum*, *Fusarium graminearum*, *Rhizopus stolonifer* and *Aspergillus nidulans*. All untreated CFSs showed various levels of inhibitory activity against the tested fungi (Figure 2), but the experimental data indicates that organic acid production was the most significant factor affecting fungal growth since after pH adjustment of the CFSs, the average growth zone reduction was 67% for *P. chrysogenum*, 69.4% for *F. graminearum*, 77.4% for *Rh. stolonifer* and 72.1% for *A. nidulans*. Muthusamy et al. [40] studied the antifungal activity of *Lb. plantarum* strains against *A. clavatus*, *A. flavus*, *P. chrysogenum* and *F. oxysporum* and attributed their high inhibitory effect mostly to the production of acidic compounds such as organic acids. The same conclusion was made by Russo et al. [88] after studying the antifungal activity of *Lb. plantarum* isolates. The main organic acids to which the antifungal effect of LAB was attributed by some studies, are lactic, acetic and phenyllactic acids [89,90]. A study on the active compounds produced by *Lb. plantarum* [91] revealed another group of pH lowering substances—3-hydroxy fatty acids, which also had an inhibitory effect against fungi.

It is interesting to note that for almost all LAB strains, catalase treatment was found to be the second important factor involved in antifungal activity against the test fungi. Results show that *P. chrysogenum* was affected by H_2_O_2_ at the lowest level compared to the other three fungi species. The average decrease of the clear zones compared to controls was 32.7%, while it was much higher for *F. graminearum* (49.5%), followed by *Rh. stolonifer* (51.1%) and *A. nidulans* (55.3%), which had the highest susceptibility to this compound. These observations are in agreement with other studies on antimicrobial activity of LAB, where inhibition was attributed to the highest extent to organic acid and H_2_O_2_ production [63].

Strain *E. faecium* 12R226 had activity against *P. chrysogenum* and *F. graminearum*, with zones of 31 and 34 mm, respectively (Figure 2A,B). However, it had a limited effect against *Rh. stolonifer* and *A. nidulans* (Figure 2C,D), which is in agreement with the relatively rare reports for antifungal activity of enterococci, especially isolated from sourdoughs [92]. The activity towards *Penicillium* spp. was totally eliminated after pH adjustment, while the CFS was still active after boiling, trypsin and catalase treatment. These observations clearly show that the antifungal activity of *E. faecium* 12R226 is mostly attributed to organic acid production. The presence of heat- and trypsin-sensitive components is also indicated by approximately 21.7 to 30.6% average clear zone reductions after the respective treatments of the LAB supernatants. However, these effects were at a much lower level compared to pH and H_2_O_2_ production.

The activity of almost all LAB strains against *F. graminearum* was higher compared to the other test fungi, with clear zones within 30 to 36 mm (Figure 2B). Belguesmia et al. [93] reported antifungal activity of *E. durans* isolated from Mongolian cheese. In our study, strain *E. durans* 09B374 was active against all tested fungi, with the highest activity (40 mm zone) towards *Aspergillus* spp. (Figure 2C).

Apart from the observed susceptibility trends for the test fungi, the antifungal activity of the tested sourdough LAB was also strain-dependent. Results show that several strains were noticeably more effective—*Pediococcus pentosaceus* 12R2192 against *P. chrysogenum* and *Rh. stolonifer*, *Lactobacillus brevis* 04P3167 against *Rh. stolonifer*, *Lactobacillus plantarum* 08B217 against *P. chrysogenum* and *F. graminearum*, *Lb. plantarum* 08B212 against *Rh. stolonifer*, *Enterococcus faecium* 12R232 against *P. chrysogenum* and *Rh. stolonifer*, *E. faecium* 12R226 against *F. graminearum*, and *E. durans* 09B374 against *A. nidulans*. *Lactobacillus brevis* 01M22 was the most effective strain, showing high results against three of the test fungi: *Fusarium* sp., *Rhizopus* sp. and *Aspergillus* sp. These results indicate potential candidates for the development of starter cultures with good biopreservation capacity for sourdoughs or other food products.

Other studies have also reported the production of antibacterial and antifungal components by LAB from sourdoughs that can improve the safety and shelf life of final products [11]. Results in Figure 1 and Figure 2 clearly show that the isolated LAB from Bulgarian sourdoughs are effective against the tested bacterial pathogens and fungi representatives, and their inhibitory activity is due to different mechanisms, which is important to take into account when selecting LAB strains for commercial sourdough starter cultures.

## 4. Conclusions

Twelve samples of typical Bulgarian sourdoughs produced without the addition of commercial yeast were collected from different locations in Bulgaria and subjected to physico-chemical and microbial characterization. It was found that pH (3.58–5.11), TTA (5.2–12.4) and dry matter content (33.01–58.16%) of the samples were not directly correlated, which could be attributed to the flour types used, diversity of the sourdough microbiota and the baker’s practices. The LAB counts ranged from 1.4 × 10^4^ to 9.7 × 10^9^ cfu/g, and yeast counts in the sourdoughs varied from 1.0 × 10^4^ to 5.3 × 10^9^ cfu/g, with ratios of 100:1 and 10:1between the two microbial groups in the samples. Molecular identification of 167 LAB isolates and 106 yeast strains revealed the diversity of these groups in Bulgarian sourdoughs. Based on the results for amylolytic and proteolytic properties and acid production capacity, 11 LAB strains were selected and tested for antibacterial and antifungal activity. Results from the study showed that the strains were effective against the tested bacterial pathogens and fungi based on different mechanisms, which is important when selecting LAB strains for commercial sourdough starter cultures. The strains *Lb. brevis* 01M22, *Lb. brevis* 07B198, *Lb. plantarum* 08B217 and *P. pentosaceus* 12R2192 were most active against both the tested bacteria and fungi. The other LAB strains with the most pronounced antifungal activity were *Lb. brevis* 04P3167, *Lb. plantarum* 08B212, *Enterococcus faecium* 12R232, *E. faecium* 12R226 and *E. durans* 09B374, which makes them good candidates for the development of active starter cultures with good biopreservation capacity for sourdoughs or other food products.

## Figures and Tables

**Table 1:** Ingredients and preparation methods of Bulgarian sourdoughs (no baker’s yeast used).

Sample Code	Depository	Flour Origin	% NaCl per Backslopping	% Sourdough Used in a Backslopping	No. of Backsloppings	Time and Temperature of Backslopping
01M	Bakery Art1, Plovdiv	*Triticum* *aestivum*,* white*	1.5	20	3	24 h/22 °C
02P1	Bakery The Bread, Plovdiv	*Secale cereale*,* wholegrain*	2.0	20	3	9 h/28 °C
03P2	Bakery Art 2, Plovdiv	*T. aestivum*,* white*	2.0	20	3	9 h/28 °C
04P3	Bakery Art 3, Plovdiv	*T. aestivum*,* white*	2.0	20	3	9 h/28 °C
05S	Bakery “8”, Smolyan	*T. aestivum*,* white*	1.5	25	3	18 h/25 °C
06SE	Homemade, Selcha	*T. aestivum/Cicer arietinum (90/10%)*	1.5	30	1	18 h/22 °C
07B1	Bakery Samun 1, Bansko	*T. aestivum*,* white*	1.5	25	3	18 h/25 °C
08B2	Bakery Samun 2, Bansko	*T. aestivum*,* wholegrain*	1.5	25	3	18 h/22 °C
09B3	Bakery Samun 3, Bansko	*Triticum monococcum*, *wholegrain*	1.5	25	3	18 h/22 °C
10B4	Bakery Samun 4, Bansko	*Secale cereale*,* wholegrain*	1.5	25	3	18 h/22 °C
11R1	Bakery Kusi 1, Ruse	*T. aestivum*,* white*	2.0	30	7	24 h/30 °C
12R2	Bakery Kusi 2, Ruse	*T. aestivum*,* white*	2.0	30	7	24 h/24 °C

**Table 2:** Physico-chemical and microbiological characterization of Bulgarian sourdoughs.

Sample	pH	TTA, mL 0.1 NaOH	Dry Matter, %	MRS		MR 5		M 17		M 17 G		SA		ME	
				TVC, cfu/mL	No. of Isolates	TVC, cfu/mL	No. of Isolates	TVC, cfu/mL	No. of Isolates	TVC, cfu/mL	No. of Isolates	TVC, cfu/mL	No. of Isolates	TVC, cfu/mL	No. of Isolates
01M	3.78	9.4	53.10	3.2 × 10^5^	4	6.1 × 10^5^	5	7.0 × 10^5^	5	2.1 × 10^5^	3	4.2 × 10^8^	4	5.4 × 10^8^	3
02P1	3.57	11.6	36.20	9.2 × 10^5^	7	4.4 × 10^5^	5	4.3 × 10^5^	4	7.9 × 10^5^	6	6.5 × 10^10^	5	6.8 × 10^10^	5
03P2	3.84	9.8	40.30	2.6 × 10^5^	3	2.7 × 10^5^	3	2.7 × 10^5^	3	4.8 × 10^5^	4	6.3 × 10^9^	5	1.1 × 10^10^	5
04P3	3.60	11.4	37.14	2.8 × 10^4^	3	2.0 × 10^5^	3	1.1 × 10^5^	7	1.1 × 10^5^	2	6.6 × 10^10^	5	7.0 × 10^10^	5
05S	3.96	10.4	58.16	3.1 × 10^12^	4	2.5 × 10^12^	4	4.2 × 10^11^	4	2.3 × 10^12^	3	5.3 × 10^9^	5	5.1 × 10^8^	5
06SE	5.11	5.2	55.21	1.7 × 10^4^	8	1.4 × 10^4^	7	3.3 × 10^5^	5	1.6 × 10^4^	7	1.0 × 10^4^	5	2.1 × 10^4^	4
07B1	3.61	12.0	42.02	2.7 × 10^11^	3	3.6 × 10^11^	4	9.9 × 10^8^	7	9.2 × 10^9^	6	6.6 × 10^10^	5	8.0 × 10^9^	4
08B2	4.07	8.1	39.39	1.8 × 10^11^	2	3.9 × 10^11^	4	2.9 × 10^10^	4	8.3 × 10^10^	6	3.5 × 10^9^	4	2.1 × 10^9^	4
09B3	4.17	8.3	43.50	2.0 × 10^11^	2	1.5 × 10^11^	7	9.8 × 10^11^	6	2.6 × 10^11^	3	6.4 × 10^9^	5	4.2 × 10^6^	4
10B4	3.58	12.4	33.01	9.7 × 10^11^	7	9.9 × 10^11^	6	8.5 × 10^8^	6	3.1 × 10^9^	4	7.4 × 10^8^	5	1.0 × 10^10^	4
11R1	3.62	10.2	49.86	3.2 × 10^7^	4	1.8 × 10^11^	2	3.5 × 10^8^	4	1.7 × 10^8^	2	9.8 × 10^8^	5	9.4 × 10^9^	4
12R2	3.64	10.0	53.18	2.1 × 10^8^	2	1.0 × 10^11^	8	2.6 × 10^8^	3	3.8 × 10^8^	4	3.4 × 10^9^	2	1.9 × 10^9^	3
				Total isolates from LAB media: 215								Total yeast isolates: 106			

## Data Availability

Not applicable.

## Supplementary Materials

The following are available online at https://www.mdpi.com/article/10.3390/microorganisms9071346/s1: Figure S1: Phylogenetic tree of LAB from Bulgarian sourdoughs, based on partial 16S rDNA sequence analysis; Figure S2: Phylogenetic tree of yeast from Bulgarian sourdoughs, based on partial sequences analysis of ITS1-5.8S-ITS2 region; Table S1: Molecular identification of LAB from Bulgarian sourdoughs by partial 16S rDNA sequence analysis; Table S2: Molecular identification of yeast from Bulgarian sourdoughs by partial sequences analysis of ITS1-5.8S-ITS2 region; Table S3: Amylolytic, proteolytic activity and acid-producing capacity of lactic acid bacteria isolated from sourdoughs.
