# Supplementary material for: Isolation and Characterization of Lactic Acid Bacteria and Yeasts from Typical Bulgarian Sourdoughs
Source: Microorganisms. 2021 Jun 22;9(7):1346. doi: 10.3390/microorganisms9071346 (PMC8306846; doi:10.3390/microorganisms9071346)
Supplement: Supplementary file 1 [file microorganisms-09-01346-s001.zip › Table S1.pdf]

Table S1. Molecular identification of LAB from Bulgarian sourdoughs by partial 16S rDNA sequence analysis.

№	Sourdough	Isolate	Closest relative (identity, %)	GenBank accession no.
**1**	01M	01M02	*Lactobacillus brevis* (100.00)	MW685433
		01M06	*Lactobacillus brevis* (100.00)	MW685432
		01M09	*Lactobacillus plantarum* (99.43)	MW774565
		01M11	*Lactobacillus plantarum* (100.00)	MW682229
		01M12	*Lactobacillus plantarum* (99.23)	MW682230
		01M18	*Lactobacillus plantarum* (100.00)	MW682231
		01M19	*Lactobacillus brevis* (99.66)	MW685431
		01M20	*Lactobacillus plantarum* (100.00)	MW682225
		01M21	*Lactobacillus brevis* (99.47)	MW685439
		01M22	*Lactobacillus brevis* (99.43)	MW685430
		01M24	*Lactobacillus plantarum* (99.56)	MW682227
		01M25	*Lactobacillus plantarum* (100.00)	MW682233
		01M26	*Lactobacillus plantarum* (100.00)	MW682226
		01M28	*Lactobacillus plantarum* (100.00)	MW682234
		01M29	*Lactobacillus brevis* (100.00)	MW685429
		01M30	*Lactobacillus plantarum* (97.95)	MW682235
**2**	02P1	02P103	*Pediococcus pentosaceus* (100.00)	MW683145
		02P104	*Pediococcus acidilactici* (99.25)	MW683129
		02P108	*Pediococcus acidilactici* (100.00)	MW683131
		02P109	*Lactobacillus plantarum* (100.00)	MW682236
		02P111	*Pediococcus pentosaceus* (99.47)	MW683146
		02P113	*Pediococcus pentosaceus* (100.00)	MW683176
		02P115	*Pediococcus pentosaceus* (99.91)	MW683177
		02P116	*Lactobacillus plantarum* (98.89)	MW682237
		02P117	*Lactobacillus plantarum subsp. plantarum* (98.91)	MW682281
		02P118	*Lactobacillus brevis* (98.37)	MW685437
		02P119	*Lactobacillus plantarum* (100.00)	MW682238
		02P120	*Lactobacillus plantarum* (100.00)	MW682239
		02P121	*Lactobacillus plantarum* (100.00)	MW682240
**3**	03P2	03P226	*Pediococcus parvulus* (100.00)	MW694895
		03P234	*Pediococcus pentosaceus* (99.92)	MW774567
		03P2102	*Pediococcus pentosaceus* (99.25)	MW683147
		03P2187	*Pediococcus acidilactici* (99.73)	MW683132
		03P2201	*Pediococcus pentosaceus* (100.00)	MW683148
		03P2218	*Pediococcus pentosaceus* (99.72)	MW683178
		03P2224	*Lactobacillus plantarum* (99.82)	MW682241
		03P2233	*Lactobacillus plantarum* (100.00)	MW682242
		03P226	*Lactobacillus brevis* (100.00)	MW685427
**4**	04P3	04P306	*Pediococcus pentosaceus* (100.00)	MW683179
		04P317	*Pediococcus pentosaceus* (99.51)	MW683149
		04P325	*Pediococcus pentosaceus* (99.83)	MW683150
		04P329	*Pediococcus pentosaceus* (99.22)	MW683180
		04P347	*Pediococcus pentosaceus* (100.00)	MW683181
		04P394	*Lactobacillus plantarum* (99.25)	MW774566
		04P3107	*Lactobacillus plantarum* (100.00)	MW682224
		04P3167	*Lactobacillus brevis* (100.00)	MW685426
		04P3188	*Lactobacillus plantarum* (99.68)	MW682223

		04P3197	*Lactobacillus plantarum* (100.00)	MW682243
		04P201	*Lactobacillus plantarum* (97.82)	MW682228
**5**	05S	05S04	*Lactobacillus brevis* (100.00)	MW685425
		05S16	*Lactobacillus plantarum* (100.00)	MW682244
		05S27	*Pediococcus acidilactici* (99.86)	MW683133
		05S29	*Lactobacillus plantarum* (100.00)	MW682245
		05S76	*Pediococcus pentosaceus* (99.06)	MW683182
		05S88	*Lactobacillus plantarum* (99.57)	MW682246
		05S103	*Lactobacillus plantarum* (99.58)	MW682222
		05S148	*Lactobacillus plantarum* (100.00)	MW682247
		05S168	*Lactobacillus brevis* (100.00)	MW685424
		05S178	*Lactobacillus brevis* (100.00)	MW685423
		05S179	*Lactobacillus plantarum* (99.61)	MW682248
		05S186	*Lactobacillus plantarum* (100.00)	MW682249
		05S189	*Lactobacillus plantarum* (99.26)	MW682250
		05S197	*Lactobacillus plantarum subsp. plantarum* (99.16)	MW682221
**6**	06SE	06SE12	*Pediococcus pentosaceus* (99.84)	MW683183
		06SE17	*Pediococcus pentosaceus* (99.06)	MW683152
		06SE22	*Pediococcus pentosaceus* (100.00)	MW683153
		06SE27	*Pediococcus pentosaceus* (100.00)	MW683184
		06SE75	*Pediococcus acidilactici* (100.00)	MW683134
		06SE78	*Pediococcus pentosaceus* (100.00)	MW683185
		06SE92	*Pediococcus acidilactici* (100.00)	MW683135
		06SE98	*Pediococcus acidilactici* (100.00)	MW683136
		06SE99	*Pediococcus acidilactici* (100.00)	MW683137
		06SE107	*Pediococcus acidilactici* (100.00)	MW683138
		06SE128	*Pediococcus pentosaceus* (100.00)	MW683186
		06SE157	*Pediococcus pentosaceus* (100.00)	MW683187
		06SE173	*Pediococcus pentosaceus* (100.00)	MW683188
		06SE204	*Pediococcus pentosaceus* (100.00)	MW683189
		06SE234	*Pediococcus acidilactici* (100.00)	MW683139
		06SE247	*Pediococcus pentosaceus* (100.00)	MW683190
		06SE251	*Pediococcus pentosaceus* (100.00)	MW683154
		06SE258	*Pediococcus pentosaceus* (99.90)	MW683155
		06SE269	*Lactobacillus brevis* (97.83)	MW685422
		06SE274	*Pediococcus pentosaceus* (99.05)	MW683156
		06SE279	*Enterococcus faecium* (99.25)	MW682282
		06SE284	*Pediococcus pentosaceus* (100.00)	MW683191
		06SE288	*Pediococcus acidilactici* (100.00)	MW683140
		06SE294	*Pediococcus acidilactici* (100.00)	MW683141
		06SE297	*Pediococcus pentosaceus* (100.00)	MW683192
		06SE342	*Pediococcus acidilactici* (100.00)	MW683142
**7**	07B1	07B110	*Lactobacillus plantarum* (100.00)	MW682251
		07B126	*Lactobacillus plantarum* (100.00)	MW682252
		07B144	*Lactobacillus plantarum* (100.00)	MW682253
		07B163	*Lactobacillus plantarum* (100.00)	MW682254
		07B169	*Pediococcus pentosaceus* (98.98)	MW683193
		07B181	*Pediococcus pentosaceus* (98.61)	MW683194
		07B187	*Lactobacillus plantarum* (99.86)	MW682255
		07B198	*Lactobacillus brevis* (99.48)	MW685435

		07B1109	*Pediococcus pentosaceus* (99.20)	MW683195
		07B1111	*Pediococcus pentosaceus* (98.81)	MW683196
		07B1119	*Pediococcus pentosaceus* (99.83)	MW683157
		07B1138	*Lactobacillus plantarum* (100.00)	MW682256
		07B1139	*Lactobacillus plantarum* (100.00)	MW682257
		07B1168	*Lactobacillus plantarum* (100.00)	MN251169.1
		07B1187	*Enterococcus faecium* (100.00)	MW682287
		07B1188	*Pediococcus pentosaceus* (100.00)	MW683158
**8**	08B2	08B201	*Lactobacillus brevis* (98.51)	MW685428
		08B212	*Lactobacillus plantarum* (100.00)	MW682259
		08B217	*Lactobacillus plantarum* (99.67)	MW682260
		08B225	*Lactobacillus brevis* (99.36)	MW685421
		08B228	*Pediococcus pentosaceus* (100.00)	MW683159
		08B238	*Lactobacillus plantarum* (100.00)	MW682261
		08B262	*Pediococcus pentosaceus* (97.20)	MW683160
		08B268	*Pediococcus pentosaceus* (99.82)	MW683161
		08B277	*Pediococcus pentosaceus* (98.05)	MW683162
		08B279	*Pediococcus pentosaceus* (99.80)	MW683163
		08B285	*Pediococcus pentosaceus* (99.15)	MW683197
		08B291	*Lactobacillus brevis* (99.11)	MW685438
		08B296	*Pediococcus pentosaceus* (100.00)	MW683198
		08B297	*Pediococcus pentosaceus* (99.57)	MW683199
**9**	09B3	09B307	*Lactobacillus plantarum* (100.00)	MW682262
		09B316	*Lactobacillus plantarum* (100.00)	MW682263
		09B324	*Lactobacillus plantarum* (100.00)	MW682264
		09B357	*Lactobacillus plantarum* (100.00)	MW682265
		09B374	*Enterococcus durans* (99.75)	MW682284
		09B378	*Pediococcus pentosaceus* (99.83)	MW683164
		09B383	*Pediococcus pentosaceus* (99.63)	MW683165
		09B391	*Pediococcus pentosaceus* (99.66)	MW683166
		09B3105	*Lactobacillus brevis* (100.00)	MW685420
		09B3154	*Pediococcus pentosaceus* (99.57)	MW683167
		09B3184	*Pediococcus pentosaceus* (99.72)	MW683168
**10**	10B4	10B412	*Lactobacillus plantarum* (100.00)	MW682266
		10B426	*Lactobacillus plantarum* (100.00)	MW682267
		10B429	*Pediococcus pentosaceus* (99.30)	MW683170
		10B447	*Lactobacillus plantarum* (100.00)	MW682268
		10B458	*Lactobacillus plantarum* (100.00)	MW682269
		10B487	*Lactobacillus plantarum* (100.00)	MW682270
		10B4101	*Lactobacillus plantarum* (100.00)	MW682271
		10B4116	*Lactobacillus plantarum* (100.00)	MW682272
		10B4122	*Lactobacillus plantarum* (100.00)	MW682273
		10B4128	*Lactobacillus plantarum* (100.00)	MW682274
		10B4147	*Lactobacillus plantarum* (100.00)	MW682275
		10B4159	*Lactobacillus plantarum* (100.00)	MW682276
		10B4181	*Lactobacillus plantarum* (100.00)	MW682277
		10B4227	*Lactobacillus plantarum* (100.00)	MW682278
		10B4261	*Lactobacillus plantarum* (100.00)	MW682279
		10B4267	*Lactobacillus plantarum* (100.00)	MW682280
		10B280	*Pediococcus pentosaceus* (100.00)	MW683169
**11**	11R1	11R115	*Pediococcus pentosaceus* (100.00)	MW683200

		11R161	*Pediococcus pentosaceus* (99.46)	MW683171
		11R187	*Pediococcus pentosaceus* (99.84)	MW683172
		11R1102	*Pediococcus pentosaceus* (100.00)	MW683173
		11R1131	*Lactobacillus plantarum subsp. plantarum* (99.63)	MW682219
		11R1143	*Lactobacillus brevis* (97.57)	MW685436
		11R1149	*Lactobacillus brevis* (100.00)	MW685419
		11R1154	*Pediococcus acidilactici* (98.61)	MW683143
**12**	12R2	12R204	*Enterococcus faecium* (100.00)	MW682286
		12R226	*Enterococcus faecium* (100.00)	MW682285
		12R232	*Enterococcus faecium* (99.07)	MW682283
		12R238	*Lactobacillus plantarum subsp. plantarum* (99.77)	MW682220
		12R287	*Lactobacillus brevis* (98.41)	MW685418
		12R298	*Pediococcus pentosaceus* (99.66)	MW683174
		12R2104	*Pediococcus pentosaceus* (99.59)	MW683201
		12R2121	*Pediococcus acidilactici* (99.81)	MW683144
		12R2162	*Lactobacillus brevis* (99.59)	MW685434
		12R2169	*Pediococcus acidilactici* (98.85)	MW683130
		12R2187	*Pediococcus pentosaceus* (99.17)	MW683202
		12R2192	*Pediococcus pentosaceus* (100.00)	MW683175
