# Supplementary material for: Isolation and Characterization of Lactic Acid Bacteria and Yeasts from Typical Bulgarian Sourdoughs
Source: Microorganisms. 2021 Jun 22;9(7):1346. doi: 10.3390/microorganisms9071346 (PMC8306846; doi:10.3390/microorganisms9071346)
Supplement: Supplementary file 1 [file microorganisms-09-01346-s001.zip › Table S2.pdf]

Table S2. Molecular identification of yeast from Bulgarian sourdoughs by partial sequences analysis of ITS1-5.8S-ITS2 region.

№	Sourdough	Isolate	Closest relative (identity, %)	GenBank accession no.
1	01M	01M06	*Saccharomyces cerevisiae* (100.00)	MW756304
		01M28	*Saccharomyces cerevisiae* (100.00)	MW756303
		01M81	*Saccharomyces cerevisiae* (100.00)	MW756299
		01M103	*Saccharomyces cerevisiae* (100.00)	MW756228
		01M148	*Kazachstania barnettii* (100.00)	MW756315
		01M173	*Saccharomyces cerevisiae* (100.00)	MW756229
		01M184	*Saccharomyces cerevisiae* (100.00)	MW756230
2	02P1	02P112	*Pichia fermentans* (100.00)	MW756305
		02P141	*Kazachstania barnettii* (100.00)	MW756227
		02P152	*Saccharomyces cerevisiae* (100.00)	MW774571
		02P167	*Saccharomyces cerevisiae* (100.00)	MW756231
		02P185	*Saccharomyces cerevisiae* (100.00)	MW756232
		02P1102	*Saccharomyces cerevisiae* (100.00)	MW756233
		02P1127	*Saccharomyces cerevisiae* (100.00)	MW756234
		02P1188	*Saccharomyces cerevisiae* (100.00)	MW756288
3	03P2	03P208	*Kluyveromyces marxianus* (100.00)	MW756309
		03P218	*Kazachstania barnettii* (100.00)	MW756319
		03P222	*Saccharomyces cerevisiae* (100.00)	MW756289
		03P297	*Saccharomyces cerevisiae* (100.00)	MW756224
		03P2101	*Saccharomyces cerevisiae* (100.00)	MW756298
		03P2127	*Saccharomyces cerevisiae* (100.00)	MW756318
		03P2143	*Saccharomyces cerevisiae* (100.00)	MW756290
		03P2187	*Saccharomyces cerevisiae* (100.00)	MW756291
		03P2207	*Saccharomyces cerevisiae* (100.00)	MW756292
		03P2211	*Saccharomyces cerevisiae* (100.00)	MW756312
		03P2217	*Saccharomyces cerevisiae* (100.00)	MW756293
		03P2221	*Saccharomyces cerevisiae* (100.00)	MW756294
4	04P3	04P309	*Yarrowia lipolytica* (100.00)	MW756276
		04P327	*Saccharomyces cerevisiae* (100.00)	MW774572
		04P338	*Saccharomyces cerevisiae* (100.00)	MW756296
		04P310	*Kluyveromyces marxianus* (100.00)	MW756308
		04P354	*Saccharomyces cerevisiae* (100.00)	MW756295
		04P387	*Saccharomyces cerevisiae* (100.00)	MW756297
		04P391	*Saccharomyces cerevisiae* (98.00)	MW756236
		04P3141	*Saccharomyces cerevisiae* (100.00)	MW756237
		04P3154	*Saccharomyces cerevisiae* (100.00)	MW756238
		04P3169	*Saccharomyces cerevisiae* (100.00)	MW756239
		04P3176	*Saccharomyces cerevisiae* (100.00)	MW756240
5	05S	05S01	*Saccharomyces cerevisiae* (100.00)	MW756241
		05S04	*Saccharomyces cerevisiae* (100.00)	MW756242
		05S18	*Saccharomyces cerevisiae* (100.00)	MW756243
		05S28	*Saccharomyces cerevisiae* (100.00)	MW756287
		05S34	*Saccharomyces cerevisiae* (100.00)	MW756316
		05S57	*Saccharomyces cerevisiae* (100.00)	MW756317
		05S59	*Saccharomyces cerevisiae* (100.00)	MW756244
		05S79	*Saccharomyces cerevisiae* (100.00)	MW756212
		05S91	*Saccharomyces cerevisiae* (100.00)	MW756213
		05S141	*Saccharomyces cerevisiae* (100.00)	MW756214
		05S173	*Saccharomyces cerevisiae* (99.23)	MW756245
6	06SE	06SE07	*Saccharomyces cerevisiae* (100.00)	MW756246
		06SE28	*Kazachstania humilis* (100.00)	MW756310
		06SE31	*Kluyveromyces marxianus* (100.00)	MW756307
		06SE58	*Kazachstania barnettii* (100.00)	MW756225

		06SE91	*Saccharomyces cerevisiae* (100.00)	MW756215
		06SE107	*Saccharomyces cerevisiae* (100.00)	MW756247
		06SE127	*Saccharomyces cerevisiae* (100.00)	MW756314
		06SE142	*Saccharomyces cerevisiae* (100.00)	MW756248
		06SE151	*Saccharomyces cerevisiae* (100.00)	MW756223
**7**	07B1	07B104	*Saccharomyces cerevisiae* (100.00)	MW756249
		07B141	*Kluyveromyces marxianus* (100.00)	MW756306
		07B162	*Pichia fermentans* (100.00)	MW756302
		07B192	*Saccharomyces cerevisiae* (100.00)	MW756286
		07B1100	*Saccharomyces cerevisiae* (100.00)	MW756250
		07B1121	*Saccharomyces cerevisiae* (100.00)	MW756251
		07B1131	*Saccharomyces cerevisiae* (100.00)	MW756252
		07B1138	*Saccharomyces cerevisiae* (100.00)	MW756285
8	08B2	08B209	*Saccharomyces cerevisiae* (100.00)	MW756253
		08B210	*Saccharomyces cerevisiae* (100.00)	MW756254
		08B217	*Saccharomyces cerevisiae* (100.00)	MW756284
		08B221	*Saccharomyces cerevisiae* (100.00)	MW756255
		08B228	*Saccharomyces cerevisiae* (100.00)	MW756256
		08B247	*Saccharomyces cerevisiae* (100.00)	MW756257
		08B252	*Saccharomyces cerevisiae* (100.00)	MW756258
		08B274	*Saccharomyces cerevisiae* (100.00)	MW756259
9	09B3	09B305	*Saccharomyces cerevisiae* (100.00)	MW756260
		09B317	*Saccharomyces cerevisiae* (100.00)	MW756261
		09B328	*Saccharomyces cerevisiae* (100.00)	MW756221
		09B339	*Yarrowia lipolytica* (100.00)	MW756277
		09B351	*Kazachstania barnettii* (100.00)	MW756226
		09B357	*Saccharomyces cerevisiae* (100.00)	MW756279
		09B374	*Saccharomyces cerevisiae* (100.00)	MW756280
		09B379	*Saccharomyces cerevisiae* (100.00)	MW756281
		09B388	*Saccharomyces cerevisiae* (100.00)	MW774573
10	10B4	10B411	*Saccharomyces cerevisiae* (100.00)	MW756283
		10B417	*Saccharomyces cerevisiae* (100.00)	MW756222
		10B478	*Saccharomyces cerevisiae* (98.63)	MW756216
		10B482	*Saccharomyces cerevisiae* (100.00)	MW756217
		10B487	*Saccharomyces cerevisiae* (100.00)	MW756218
		10B489	*Saccharomyces cerevisiae* (100.00)	MW756262
		10B495	*Saccharomyces cerevisiae* (100.00)	MW756219
		10B4102	*Saccharomyces cerevisiae* (100.00)	MW756263
		10B4111	*Saccharomyces cerevisiae* (100.00)	MW756264
11	11R1	11R102	*Saccharomyces cerevisiae* (100.00)	MW756265
		11R114	*Saccharomyces cerevisiae* (100.00)	MW756266
		11R117	*Saccharomyces cerevisiae* (99.86)	MW756300
		11R127	*Saccharomyces cerevisiae* (99.47)	MW756220
		11R139	*Saccharomyces cerevisiae* (100.00)	MW756278
		11R159	*Saccharomyces cerevisiae* (100.00)	MW756267
		11R1119	*Saccharomyces cerevisiae* (100.00)	MW756268
		11R1124	*Saccharomyces cerevisiae* (100.00)	MW756269
		11R1128	*Saccharomyces cerevisiae* (100.00)	MW756270
12	12R2	12R210	*Saccharomyces cerevisiae* (100.00)	MW756271
		12R216	*Saccharomyces cerevisiae* (100.00)	MW756272
		12R227	*Saccharomyces cerevisiae* (100.00)	MW756273
		12R239	*Saccharomyces cerevisiae* (100.00)	MW756274
		12R241	*Saccharomyces cerevisiae* (100.00)	MW756275
