# Supplementary material for: Isolation and Characterization of Lactic Acid Bacteria and Yeasts from Typical Bulgarian Sourdoughs
Source: Microorganisms. 2021 Jun 22;9(7):1346. doi: 10.3390/microorganisms9071346 (PMC8306846; doi:10.3390/microorganisms9071346)
Supplement: Supplementary file 1 [file microorganisms-09-01346-s001.zip › Table S3.pdf]

Table S3. Amylolytic, proteolytic activity and acid producing capacity of lactic acid bacteria isolated from sourdoughs.

No.	LAB strain	Amylolytic index	Protein hydrolysis index	Acid producing capacity (Δ pH)
1	*Pediococcus pentosaceus* 02P103	3.83 ± 0.02	4.62 ± 0.04	1.62 ± 0.03
2	*Pediococcus pentosaceus* 02P111	3.61 ± 0.06	4.48± 0.03	1.51 ± 0.03
3	*Pediococcus pentosaceus* 02P113	2.57 ± 0.11	4.24 ± 0.02	1.38 ± 0.02
4	*Pediococcus pentosaceus* 02P115	4.02 ± 0.08	4.68 ± 0.02	1.47 ± 0.03
5	*Pediococcus pentosaceus* 03P234	3.28 ± 0.09	4.78 ± 0.04	1.64 ± 0.02
6	*Pediococcus pentosaceus* 03P2102	3.69 ± 0.10	3.98 ± 0.04	1.22 ± 0.02
7	*Pediococcus pentosaceus* 03P2201	4.67 ± 0.04	4.98 ± 0.02	1.41 ± 0.03
8	*Pediococcus pentosaceus* 03P2218	2.94 ± 0.06	4.38 ± 0.06	1.29 ± 0.03
9	*Pediococcus pentosaceus* 04P306	3.57 ± 0.03	4.14 ± 0.04	1.74 ± 0.02
10	*Pediococcus pentosaceus* 04P317	3.95 ± 0.06	4.74 ± 0.06	1.35 ± 0.02
11	*Pediococcus pentosaceus* 04P325	4.68 ± 0.05	4.28 ± 0.04	1.63 ± 0.03
12	*Pediococcus pentosaceus* 04P329	2.05 ± 0.04	3.88 ± 0.02	1.62 ± 0.02
13	*Pediococcus pentosaceus* 04P347	2.66 ± 0.06	4.73 ± 0.04	1.44 ± 0.02
14	*Pediococcus pentosaceus* 05S76	4.53 ± 0.09	4.83 ± 0.02	1.46 ± 0.02
15	*Pediococcus pentosaceus* 06SE12	4.71 ± 0.06	4.37 ± 0.04	1.23 ± 0.03
16	*Pediococcus pentosaceus* 06SE17	3.28 ± 0.04	3.68 ± 0.05	1.46 ± 0.03
17	*Pediococcus pentosaceus* 06SE22	3.14 ± 0.08	4.58 ± 0.02	1.63 ± 0.02
18	*Pediococcus pentosaceus* 06SE27	3.32 ± 0.12	4.69 ± 0.03	1.66 ± 0.02
19	*Pediococcus pentosaceus* 06SE128	3.68 ± 0.04	4.25 ± 0.04	1.54 ± 0.03
20	*Pediococcus pentosaceus* 06SE157	4.12 ± 0.08	4.38 ± 0.03	1.26 ± 0.02
21	*Pediococcus pentosaceus* 06SE173	2.68 ± 0.06	4.88 ± 0.02	1.57 ± 0.03
22	*Pediococcus pentosaceus* 06SE204	3.04 ± 0.11	4.57 ± 0.06	1.58 ± 0.03
23	*Pediococcus pentosaceus* 06SE247	2.84 ± 0.04	4.47 ± 0.03	1.74 ± 0.03
24	*Pediococcus pentosaceus* 06SE251	4.29 ± 0.10	4.84 ± 0.06	1.68 ± 0.03
25	*Pediococcus pentosaceus* 06SE258	3.69 ± 0.02	4.83 ± 0.02	1.45 ± 0.02
26	*Pediococcus pentosaceus* 06SE274	3.27 ± 0.06	3.56 ± 0.04	1.42 ± 0.02
27	*Pediococcus pentosaceus* 06SE284	3.86 ± 0.04	4.86 ± 0.02	1.58 ± 0.02
28	*Pediococcus pentosaceus* 06SE297	3.53 ± 0.04	4.88 ± 0.04	1.62 ± 0.02
29	*Pediococcus pentosaceus* 07B169	3.19 ± 0.02	4.52 ± 0.05	1.62 ± 0.03
30	*Pediococcus pentosaceus* 07B181	2.96 ± 0.08	4.48 ± 0.08	1.32 ± 0.03
31	*Pediococcus pentosaceus* 07B1109	4.98 ± 0.05	5.12 ± 0.06	1.84 ± 0.02
32	*Pediococcus pentosaceus* 07B1111	3.39 ± 0.04	4.78 ± 0.02	1.67 ± 0.03
33	*Pediococcus pentosaceus* 07B1119	2.68 ± 0.07	4.88 ± 0.04	1.47 ± 0.02
34	*Pediococcus pentosaceus* 07B1188	3.12 ± 0.02	4.14 ± 0.05	1.47 ± 0.02
35	*Pediococcus pentosaceus* 08B228	4.18 ± 0.04	3.94 ± 0.04	1.71 ± 0.03
36	*Pediococcus pentosaceus* 08B262	3.39 ± 0.07	3.75 ± 0.05	1.43 ± 0.01
37	*Pediococcus pentosaceus* 08B268	4.25 ± 0.04	4.44 ± 0.06	1.64 ± 0.03
38	*Pediococcus pentosaceus* 08B277	3.98 ± 0.11	4.68 ± 0.04	1.54 ± 0.02
39	*Pediococcus pentosaceus* 08B279	3.29 ± 0.06	4.76 ± 0.02	1.41 ± 0.02
40	*Pediococcus pentosaceus* 08B285	3.56 ± 0.02	4.28 ± 0.04	1.72 ± 0.02
41	*Pediococcus pentosaceus* 08B296	2.83 ± 0.06	4.88 ± 0.06	1.57 ± 0.03
42	*Pediococcus pentosaceus* 08B297	3.58 ± 0.42	4.56 ± 0.04	1.53 ± 0.02
43	*Pediococcus pentosaceus* 09B378	3.44 ± 0.06	4.68 ± 0.04	1.47 ± 0.02
44	*Pediococcus pentosaceus* 09B383	3.02 ± 0.08	4.47 ± 0.04	1.37 ± 0.02

45	*Pediococcus pentosaceus* 09B391	3.87 ± 0.06	4.57 ± 0.02	1.73 ± 0.03
46	*Pediococcus pentosaceus* 09B3154	4.44 ± 0.05	4.37 ± 0.05	1.64 ± 0.02
47	*Pediococcus pentosaceus* 09B3184	3.89 ± 0.05	3.64 ± 0.05	1.58 ± 0.03
48	*Pediococcus pentosaceus* 10B429	3.65 ± 0.07	4.12 ± 0.04	1.73 ± 0.02
49	*Pediococcus pentosaceus* 10B280	3.86 ± 0.04	4.43 ± 0.02	1.46 ± 0.02
50	*Pediococcus pentosaceus* 11R115	3.62 ± 0.02	4.82 ± 0.05	1.47 ± 0.01
51	*Pediococcus pentosaceus* 11R161	4.28 ± 0.05	4.13 ± 0.07	1.56 ± 0.02
52	*Pediococcus pentosaceus* 11R187	4.68 ± 0.02	4.27 ± 0.04	1.64 ± 0.03
53	*Pediococcus pentosaceus* 11R1102	3.98 ± 0.05	4.38 ± 0.04	1.65 ± 0.02
54	*Pediococcus pentosaceus* 12R298	2.95 ± 0.03	4.87 ± 0.03	1.62 ± 0.01
55	*Pediococcus pentosaceus* 12R2104	3.74 ± 0.06	4.79 ± 0.02	1.70 ± 0.03
56	*Pediococcus pentosaceus* 12R2187	3.58 ± 0.05	5.27 ± 0.04	1.81 ± 0.01
57	*Pediococcus pentosaceus* 12R2192	5.79 ± 0.05	6.68 ± 0.02	1.89 ± 0.03
58	*Lactobacillus plantarum* 01M09	3.55 ± 0.07	4.88 ± 0.04	1.64 ± 0.02
59	*Lactobacillus plantarum* 01M11	3.68 ± 0.10	4.26 ± 0.06	1.62 ± 0.02
60	*Lactobacillus plantarum* 01M12	3.24 ± 0.06	4.79 ± 0.04	1.71 ± 0.03
61	*Lactobacillus plantarum* 01M18	3.54 ± 0.03	5.12 ± 0.06	1.78 ± 0.03
62	*Lactobacillus plantarum* 01M20	3.92 ± 0.03	5.56 ± 0.02	1.82 ± 0.03
63	*Lactobacillus plantarum* 01M24	4.21 ± 0.05	4.96 ± 0.04	1.66 ± 0.03
64	*Lactobacillus plantarum* 01M25	4.94 ± 0.04	5.68 ± 0.02	1.80 ± 0.02
65	*Lactobacillus plantarum* 01M26	3.21 ± 0.04	5.04 ± 0.05	1.84 ± 0.03
66	*Lactobacillus plantarum* 01M28	3.67 ± 0.02	4.78 ± 0.02	1.47 ± 0.02
67	*Lactobacillus plantarum* 01M30	3.85 ± 0.04	4.62 ± 0.04	1.50 ± 0.02
68	*Lactobacillus plantarum* 02P109	3.56 ± 0.04	4.94 ± 0.04	1.48 ± 0.03
69	*Lactobacillus plantarum* 02P119	3.62 ± 0.03	5.17 ± 0.03	1.76 ± 0.02
70	*Lactobacillus plantarum* 02P120	3.42 ± 0.06	5.20 ± 0.05	1.83 ± 0.03
71	*Lactobacillus plantarum* 02P121	3.86 ± 0.04	5.14 ± 0.04	1.77 ± 0.02
72	*Lactobacillus plantarum* 03P2233	3.33 ± 0.07	4.82 ± 0.02	1.41 ± 0.03
73	*Lactobacillus plantarum* 04P394	4.06 ± 0.02	4.78 ± 0.08	1.60 ± 0.03
74	*Lactobacillus plantarum* 04P3188	4.35 ± 0.04	5.08 ± 0.10	1.79 ± 0.02
75	*Lactobacillus plantarum* 04P3197	4.21 ± 0.02	5.32 ± 0.04	1.69 ± 0.03
76	*Lactobacillus plantarum* 04P201	3.88 ± 0.02	4.84 ± 0.02	1.39 ± 0.02
77	*Lactobacillus plantarum* 05S29	3.43 ± 0.06	4.66 ± 0.06	1.43 ± 0.01
78	*Lactobacillus plantarum* 05S88	3.48 ± 0.04	5.24 ± 0.02	1.78 ± 0.03
79	*Lactobacillus plantarum* 05S103	3.58 ± 0.02	4.47 ± 0.04	1.52 ± 0.02
80	*Lactobacillus plantarum* 05S148	3.34 ± 0.05	4.68 ± 0.04	1.65 ± 0.03
81	*Lactobacillus plantarum* 05S179	3.61 ± 0.05	4.66 ± 0.05	1.67 ± 0.02
82	*Lactobacillus plantarum* 05S186	3.14 ± 0.06	4.58 ± 0.06	1.67 ± 0.01
83	*Lactobacillus plantarum* 05S189	3.86 ± 0.04	4.78 ± 0.04	1.47 ± 0.03
84	*Lactobacillus plantarum* 07B110	3.32 ± 0.04	4.63 ± 0.05	1.57 ± 0.03
85	*Lactobacillus plantarum* 07B126	3.54 ± 0.02	5.00 ± 0.04	1.83 ± 0.03
86	*Lactobacillus plantarum* 07B144	3.48 ± 0.04	5.14 ± 0.06	1.82 ± 0.02
87	*Lactobacillus plantarum* 07B163	3.76 ± 0.06	4.96 ± 0.04	1.62 ± 0.02
88	*Lactobacillus plantarum* 07B187	3.62 ± 0.07	4.82 ± 0.02	1.62 ± 0.03
89	*Lactobacillus plantarum* 07B1138	3.36 ± 0.04	4.83 ± 0.02	1.56 ± 0.02
90	*Lactobacillus plantarum* 07B1139	3.56 ± 0.02	4.77 ± 0.05	1.48 ± 0.03
91	*Lactobacillus plantarum* 07B1168	3.98 ± 0.06	4.68 ± 0.04	1.52 ± 0.03
92	*Lactobacillus plantarum* 08B212	4.86 ± 0.06	5.48 ± 0.02	1.81 ± 0.02

93	*Lactobacillus plantarum* 08B217	4.59 ± 0.02	5.23 ± 0.04	1.78 ± 0.01
94	*Lactobacillus plantarum* 08B238	4.68 ± 0.06	4.96 ± 0.06	1.68 ± 0.03
95	*Lactobacillus plantarum* 09B307	3.47 ± 0.09	4.88 ± 0.05	1.55 ± 0.02
96	*Lactobacillus plantarum* 09B316	3.68 ± 0.06	4.58 ± 0.02	1.47 ± 0.03
97	*Lactobacillus plantarum* 09B324	3.38 ± 0.06	4.47 ± 0.02	1.38 ± 0.02
98	*Lactobacillus plantarum* 09B357	3.54 ± 0.07	4.69 ± 0.04	1.45 ± 0.03
99	*Lactobacillus plantarum* 10B412	3.98 ± 0.04	4.83 ± 0.06	1.39 ± 0.02
100	*Lactobacillus plantarum* 10B426	3.88 ± 0.02	4.54 ± 0.02	1.57 ± 0.03
101	*Lactobacillus plantarum* 10B447	3.52 ± 0.04	4.38 ± 0.04	1.61 ± 0.02
102	*Lactobacillus plantarum* 10B458	3.26 ± 0.02	4.55 ± 0.02	1.57 ± 0.02
103	*Lactobacillus plantarum* 10B487	3.48 ± 0.02	5.14 ± 0.06	1.78 ± 0.03
104	*Lactobacillus plantarum* 10B4101	3.44 ± 0.03	5.22 ± 0.02	1.78 ± 0.01
105	*Lactobacillus plantarum* 10B4116	3.73 ± 0.04	4.88 ± 0.04	1.62 ± 0.02
106	*Lactobacillus plantarum* 10B4122	3.84 ± 0.03	4.69 ± 0.02	1.51 ± 0.03
107	*Lactobacillus plantarum* 10B4128	3.47 ± 0.04	4.74 ± 0.02	1.48 ± 0.03
108	*Lactobacillus plantarum* 10B4147	3.12 ± 0.02	4.66 ± 0.05	1.53 ± 0.03
109	*Lactobacillus plantarum* 10B4159	2.87 ± 0.06	5.10 ± 0.04	1.76 ± 0.02
110	*Lactobacillus plantarum* 10B4181	2.24 ± 0.02	5.16 ± 0.04	1.70 ± 0.02
111	*Lactobacillus plantarum* 10B4197	2.57 ± 0.05	5.06 ± 0.02	1.69 ± 0.03
112	*Lactobacillus plantarum* 10B4227	3.23 ± 0.04	4.88 ± 0.02	1.57 ± 0.03
113	*Lactobacillus plantarum* 10B4261	3.14 ± 0.06	4.58 ± 0.02	1.49 ± 0.02
114	*Lactobacillus plantarum* 10B4267	3.28 ± 0.03	4.67 ± 0.05	1.64 ± 0.02
115	*Lactobacillus brevis* 01M06	4.24 ± 0.02	4.98 ± 0.02	1.46 ± 0.02
116	*Lactobacillus brevis* 01M19	4.83 ± 0.04	4.67 ± 0.05	1.66 ± 0.03
117	*Lactobacillus brevis* 01M21	4.47 ± 0.02	5.28 ± 0.06	1.88 ± 0.03
118	*Lactobacillus brevis* 01M22	5.23 ± 0.05	5.66 ± 0.04	1.86 ± 0.03
119	*Lactobacillus brevis* 01M29	4.82 ± 0.06	5.46 ± 0.04	1.80 ± 0.01
120	*Lactobacillus brevis* 02P116	3.88 ± 0.10	4.78 ± 0.02	1.69 ± 0.03
121	*Lactobacillus brevis* 02P118	4.57 ± 0.06	5.02 ± 0.04	1.76 ± 0.02
122	*Lactobacillus brevis* 03P2224	4.89 ± 0.04	5.10 ± 0.05	1.80 ± 0.02
123	*Lactobacillus brevis* 03P226	4.53 ± 0.02	4.86 ± 0.02	1.61 ± 0.02
124	*Lactobacillus brevis* 04P3167	5.14 ± 0.07	5.64 ± 0.02	1.88 ± 0.01
125	*Lactobacillus brevis* 05S04	4.73 ± 0.07	4.35 ± 0.06	1.32 ± 0.02
126	*Lactobacillus brevis* 05S16	4.68 ± 0.04	4.97 ± 0.04	1.53 ± 0.03
127	*Lactobacillus brevis* 05S168	4.74 ± 0.03	5.28 ± 0.05	1.70 ± 0.03
128	*Lactobacillus brevis* 05S178	4.92 ± 0.04	5.18 ± 0.04	1.72 ± 0.02
129	*Lactobacillus brevis* 06SE269	3.78 ± 0.07	5.04 ± 0.02	1.81 ± 0.01
130	*Lactobacillus brevis* 07B198	5.33 ± 0.04	5.52 ± 0.02	1.91 ± 0.02
131	*Lactobacillus brevis* 08B201	4.13 ± 0.04	4.64 ± 0.02	1.58 ± 0.03
132	*Lactobacillus brevis* 08B225	4.48 ± 0.05	4.84 ± 0.04	1.54 ± 0.01
133	*Lactobacillus brevis* 08B291	4.02 ± 0.07	4.58 ± 0.02	1.69 ± 0.02
134	*Lactobacillus brevis* 09B3105	3.88 ± 0.02	4.65 ± 0.05	1.62 ± 0.02
135	*Lactobacillus brevis* 11R1143	3.64 ± 0.03	4.84 ± 0.02	1.60 ± 0.03
136	*Lactobacillus brevis* 11R1149	3.26 ± 0.04	4.68 ± 0.02	1.67 ± 0.03
137	*Lactobacillus brevis* 12R287	4.03 ± 0.04	4.86 ± 0.04	1.38 ± 0.02
138	*Lactobacillus brevis* 12R2162	3.78 ± 0.04	4.96 ± 0.02	1.47 ± 0.01
139	*Pediococcus acidilactici* 02P104	2.57 ± 0.02	4.64 ± 0.04	1.45 ± 0.03
140	*Pediococcus acidilactici* 02P108	3.12 ± 0.04	5.03 ± 0.05	1.76 ± 0.02

141	*Pediococcus acidilactici* 03P2187	3.04 ± 0.07	5.12 ± 0.02	1.71 ± 0.03
142	*Pediococcus acidilactici* 04P3107	2.73 ± 0.04	4.84 ± 0.04	1.66 ± 0.02
143	*Pediococcus acidilactici* 05S27	3.54 ± 0.02	4.67 ± 0.06	1.70 ± 0.03
144	*Pediococcus acidilactici* 06SE75	3.32 ± 0.07	4.96 ± 0.02	1.61 ± 0.02
145	*Pediococcus acidilactici* 06SE78	3.62 ± 0.04	5.10 ± 0.02	1.74 ± 0.01
146	*Pediococcus acidilactici* 06SE92	3.83 ± 0.02	5.15 ± 0.04	1.78 ± 0.03
147	*Pediococcus acidilactici* 06SE98	2.87 ± 0.06	4.88 ± 0.02	1.66 ± 0.02
148	*Pediococcus acidilactici* 06SE99	2.39 ± 0.03	4.76 ± 0.06	1.70 ± 0.03
149	*Pediococcus acidilactici* 06SE107	3.86 ± 0.03	4.62 ± 0.04	1.45 ± 0.01
150	*Pediococcus acidilactici* 06SE234	3.23 ± 0.04	4.77 ± 0.05	1.54 ± 0.03
151	*Pediococcus acidilactici* 06SE288	2.78 ± 0.02	4.55 ± 0.02	1.53 ± 0.02
152	*Pediococcus acidilactici* 06SE294	2.56 ± 0.02	4.60 ± 0.04	1.47 ± 0.01
153	*Pediococcus acidilactici* 06SE342	2.94 ± 0.04	4.81 ± 0.02	1.48 ± 0.02
154	*Pediococcus acidilactici* 11R1154	3.28 ± 0.06	4.73 ± 0.05	1.60 ± 0.03
155	*Pediococcus acidilactici* 12R2121	3.31 ± 0.05	4.74 ± 0.02	1.58 ± 0.02
156	*Pediococcus acidilactici* 12R2169	3.68 ± 0.04	4.86 ± 0.02	1.57 ± 0.03
157	*Enterococcus faecium* 06SE279	4.64 ± 0.02	5.46 ± 0.04	1.80 ± 0.03
158	*Enterococcus faecium* 07B1187	4.58 ± 0.04	5.02 ± 0.06	1.87 ± 0.01
159	*Enterococcus faecium* 12R204	3.94 ± 0.03	5.15 ± 0.03	1.79 ± 0.02
160	*Enterococcus faecium* 12R226	4.88 ± 0.04	5.74 ± 0.04	1.80 ± 0.02
161	*Enterococcus faecium* 12R232	4.96 ± 0.02	5.88 ± 0.02	1.88 ± 0.03
162	*Lactobacillus plantarum subsp. plantarum* 02P117	3.54 ± 0.04	4.84 ± 0.06	1.61 ± 0.03
163	*Lactobacillus plantarum subsp. plantarum* 05S197	3.48 ± 0.04	4.74 ± 0.02	1.66 ± 0.02
164	*Lactobacillus plantarum subsp. plantarum* 11R1131	3.86 ± 0.04	4.61 ± 0.04	1.67 ± 0.02
165	*Lactobacillus plantarum subsp. plantarum* 12R238	3.48 ± 0.03	4.75 ± 0.03	1.48 ± 0.03
166	*Enterococcus durans* 09B374	4.42 ± 0.05	5.26 ± 0.06	1.75 ± 0.03
167	*Pediococcus parvulus 03P226*	3.28 ± 0.04	4.72 ± 0.04	1.68 ± 0.02
