# Supplementary figures and images for: Isolation and Characterization of Lactic Acid Bacteria and Yeasts from Typical Bulgarian Sourdoughs
Source: Microorganisms. 2021 Jun 22;9(7):1346. doi: 10.3390/microorganisms9071346 (PMC8306846; doi:10.3390/microorganisms9071346)

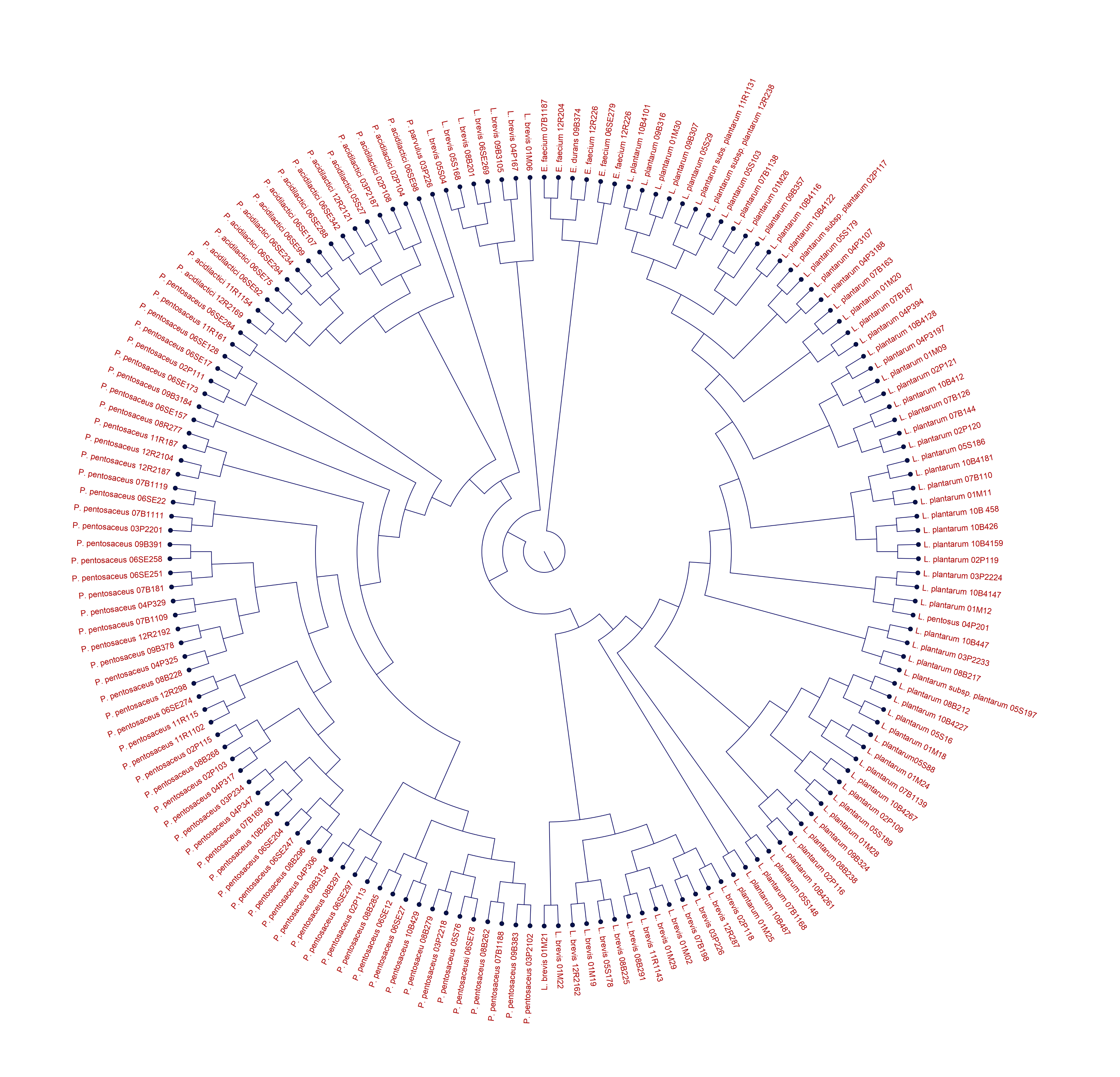

Supplement: Supplementary file 1 [file microorganisms-09-01346-s001.zip › Figure S1 LAB_Bulgarian sourdoughs.png]

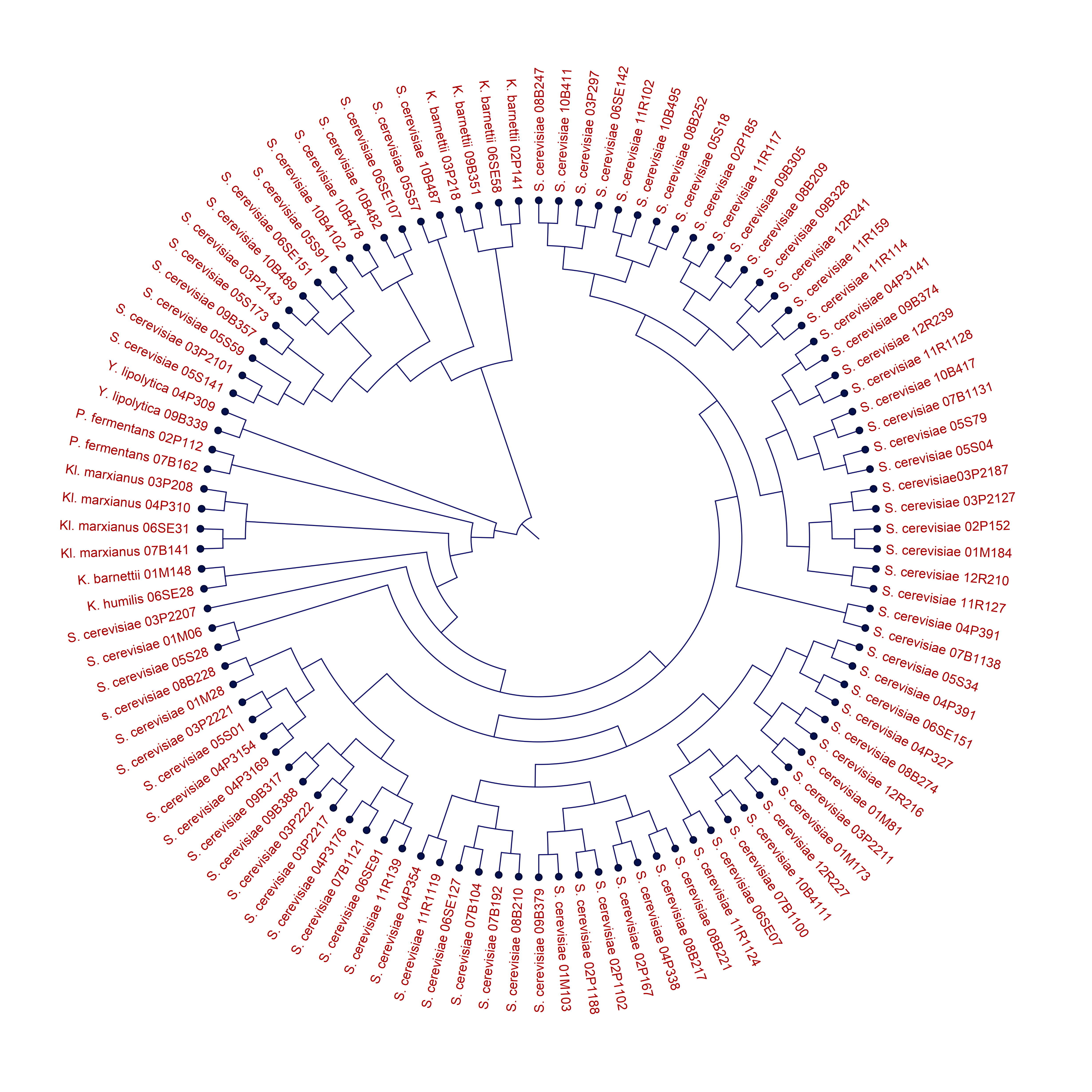

Supplement: Supplementary file 1 [file microorganisms-09-01346-s001.zip › Figure S2 Yeast_Bulgarian sourdoughs.png]
